# Sex-specific decision-making impairments and striatal dopaminergic changes after binge drinking history in rats

**DOI:** 10.3389/fphar.2023.1076465

**Published:** 2023-01-16

**Authors:** Pierre Sauton, Jerome Jeanblanc, Farid Benzerouk, Fabien Gierski, Mickael Naassila

**Affiliations:** ^1^ INSERM UMR 1247—Research Group on Alcohol & Pharmacodependences (GRAP), Université de Picardie Jules Verne, Centre Universitaire de Recherche en Santé, Amiens, France; ^2^ Université de Reims Champagne-Ardenne, Laboratoire Cognition, Santé, Société (C2S, EA6291), Reims, France

**Keywords:** binge drinking, decision making, dopamine, nucleus accumbens core, sex

## Abstract

Binge drinking (BD) is a harmful behavior for health and is a predictive factor for the development of alcohol addiction. Weak decision-making (DM) capacities could play a role in the vulnerability to BD which in turn would lead to DM impairments, thus perpetuating BD. Longitudinal preclinical studies are however lacking and necessary to understand this complex relationship. Both DM and BD are influenced by sex and involve dopamine release in the core of the nucleus accumbens, a central mechanism regulated by dopamine D2/3 autoreceptors. In this context, we used an operant self-administration procedure of BD in male and female rats, and longitudinally assessed DM capacity, memory and anxiety-like behavior. To better understand the mechanisms potentially involved in the relationship between DM and BD, *ex vivo* dopamine transmission was assessed short term after the end of the binge exposure in the core of the nucleus accumbens (NAc) using the fast-scan cyclic voltammetry (FSCV) technique and the D2/3 agonist quinpirole. We found important basal sex differences in DM, with female rats showing better performances at baseline. Choice processes were impaired exclusively in males after BD history, associated with a decrease in impulse control in both sexes, while memory and anxiety-like behavior were not affected. Our neurobiological results demonstrate that BD did not affect basal dopamine signaling in the NAc core, regardless of the sex, but reveal changes in the sensitivity to the inhibitory effects of quinpirole in females. DM impairments were neither associated with changes in basal dopamine signaling nor pre-synaptic D2 activity. Overall, our findings show that BD affects both DM processes and dopamine transmission in the core of the NAc in a sex-related manner, further suggesting that these effects may play a role in the vicious cycle leading to BD perpetuation and the early onset of AUD. Our results may inform novel strategies for therapeutic and prevention interventions.

## 1 Introduction

Binge drinking (BD) definition has yet to find an international consensus but it is characterized by an intense and episodic alcohol consumption, with recurring alternations between intense intoxication episodes and abstinence periods (Lannoy et al., 2019). BD is particularly worrying because of its health consequences (Kuntsche et al., 2017; Rolland and Naassila, 2017; Tavolacci et al., 2019) and its contribution to alcohol dependence vulnerability (Tavolacci et al., 2019).

Many factors have been associated with BD such as decision-making (DM) and impulsivity, two hallmarks of alcohol dependence. First, DM refers to a fundamental cognitive process allowing us to select a particular option among several alternatives, in order to select advantageous choices over disadvantageous one in everyday life [for review, (Ernst and Paulus, 2005)]. A recent meta-analysis showed a strong association between poor DM capacities using the Iowa Gambling Task (IGT), a tool widely accepted as a direct assessment method for measuring affective DM, and Alcohol Use Disorders (AUD) (Kovács et al., 2017). Furthermore, it has been shown that affective DM moderates the effects of associations on alcohol (Cappelli et al., 2017). Much less is known regarding BD, and the current results from the scientific literature are scarce. Previous studies have associated high and stable BD in college students with less advantageous choices in the IGT, hypersensitivity to reward and impaired reversal learning, without correlation to impulsivity or working memory capacities and academic school performances (Goudriaan et al., 2007; Johnson et al., 2008; Yoo and Kim, 2016). Impaired DM performances and response inhibition have also been reported as a predictor of future problematic use of alcohol (Goudriaan et al., 2011). Other studies have however found no relationship between BD and DM, suggesting that impairments of this cognitive function are associated with more severe forms of alcohol consumption (Bø et al., 2016; Carbia et al., 2017). Secondly, higher impulsivity scores have been associated with the maintenance and intensity of BD (Leeman et al., 2015; Adan et al., 2017), and correlated with drinking episode frequency and the number of drinks per episode (Doumas et al., 2017) (Ashenhurst et al., 2016). Overall, most previously cited studies have shown that BD is associated with impaired impulse control and DM abilities, but the link between them is poorly understood. Weak DM capacities could play a role in the vulnerability to BD, which in turn would lead to DM impairments and thus perpetuate BD. Most studies are cross-sectional and there is a clear need for further longitudinal studies. In a cross-sectional study we previously showed using a Rat Gambling Task (RGT), a protocol inspired from the IGT and described as an animal model of affective decision-making (de Visser et al., 2011), that a history of voluntary BD is associated with impairments of DM abilities in male rats (Jeanblanc et al., 2019). Longitudinal preclinical studies are lacking in the BD research field with the possibility to better control for various factors than in clinical studies (Jeanblanc et al., 2018).

Both DM and BD behaviors are influenced by sex. For instance, findings supported the subdivision of binge drinkers according to gender and personality dimensions (Gierski et al., 2017). Sex differences in IGT performances are however poorly understood, some human studies highlighting males outperforming females in gain (Evans and Hampson, 2015), while others found no difference (Hooper et al., 2004). It has been non-etheless suggested that women may use a different choice strategy, and are more sensitive than men to punishment frequency and occasional losses in the IGT long-term advantageous decks (van den Bos et al., 2013). Sex differences in DM may be attributable in part to interactions between gonadal hormones and dopamine signaling (Becker and Hu, 2008). Previous work have also shown that males and females differ in their responses to dopamine manipulations that could involve basal differences in extracellular levels of dopamine, dopamine receptor levels, and/or dopamine D2/3 autoreceptors control, all of which are modulated by estradiol (Becker and Hu, 2008).

Alteration of dopamine signaling in the ventral striatum is involved in the effects of BD and DM processes. Acute alcohol intake increases tonic dopamine concentrations in the NAc (Imperato and Di Chiara, 1986; Di Chiara and Imperato, 1988) and is involved in alcohol rewarding effects (for review, (Spanagel, 2009)). Alcohol displays biphasic effects on evoked phasic dopamine release in the NAc *in vivo* (Budygin et al., 2001; Robinson et al., 2005; Jones et al., 2006; Pelkonen et al., 2010) and *ex-vivo* (Budygin et al., 2001; Mathews et al., 2006) as well as an increase in the frequency of phasic dopamine transients (Robinson et al., 2009). Effects of chronic alcohol exposure and especially BD are much less investigated. Only a handful of studies assessed the consequences of a binge-like exposure on the dopamine mesolimbic system, but reveals possible impairments of the mesolimbic dopaminergic system, both at baseline and in response to alcohol, depending on age, dose and the withdrawal time (Pascual et al., 2009; Philpot et al., 2009; Shnitko et al., 2016; Zandy et al., 2016). Dopamine D2/3 receptors play a crucial role in both DM and alcohol reinforcing effects. Increasing dopamine release by either ventral tegmental area stimulation, or blockade of the D2/3 autoreceptors is associated with increased risky choices (Stopper and Floresco, 2015). In the same vein, the administration of a D2 receptor antagonist in pathological gamblers increased the rewarding effect of gambling and the desire to gamble (Zack and Poulos, 2007). Regarding addiction, numerous studies have shown that a low D2/3 receptor binding is associated with AUD in both animals and humans (Trifilieff and Martinez, 2014). Overall, phasic dopamine signaling in the NAc is involved in many central processes of reward and DM, and its alteration by alcohol could possibly constitute one of the main factors underlying its effects on both BD and DM capacities.

We posited that BD would decrease DM capacities associated with alterations in ventral striatum dopamine signaling and we therefore used the RGT with a longitudinal approach to investigate changes in DM capacities after a history of BD. We also explored whether BD could impact memory and anxiety-like behavior, since both could potentially interfere with DM capacities. We hypothesized that DM deficits induced by BD may be associated with changes in dopamine release potentially due to alteration in D2/3 receptors. In brain slices from the same animals, we tested the sensitivity of dopamine transmission to quinpirole (D2/3 agonist), due to the involvement of D2/3 receptors in DM. We decided to focus on the core part of the NAc due to its involvement in both DM (Sugam et al., 2012) and the approach and treatment of motivational stimuli (Dreyer et al., 2016). The NAc core plays a more significant role than the shell in behaviors regulated by cues, such as lever pressing for a reward or a cue-induced reinstatement of reward seeking (McFarland et al., 2003; Di Ciano et al., 2008). Different procedures to study BD behavior in animals are used mainly consisting in repeated passive exposures to alcohol and during adolescence (Pascual et al., 2009). More relevant models exist such as the one we recently developed based on the “happy hour” session in which the animal can freely self-administer a 20% ethanol solution during daily sessions of only 15 min (Jeanblanc et al., 2018). We used males and females with an operant self-administration procedure to evaluate inter-individual vulnerability (Jacobs et al., 2003; Jeanblanc et al., 2019).

## 2 Methods and materials

### 2.1 Reagents

Ethanol (96%) was purchased from WWR (Prolabo, Fontenay-sous-Bois, France) and diluted into tap water at a 20% concentration (v/v). NaCl, KCl, NaH_2_PO_4_, MgCl_2_, CaCl_2_, NaHCO_3_, glucose, ascorbic acid and quinpirole were purchased from Sigma-Aldrich (Saint Quentin Fallavier, France).

### 2.2 Animals

32 Long Evans rats (16 males and 16 females) were purchased from Janvier Laboratories (Le genest-Saint-Isle, France) at the age of 8 weeks. The animals were single housed in individually ventilated cage (IVC) with food and water *ad libitum* and no enrichment. The light phase started at 7:00 a.m. for 12 h. The experiments started 1 month after their arrival. Prior to all experiments, 20 of the animals were randomly assigned to a group of voluntary alcohol administration (10 males and 10 females), and the remaining 12 rats (6 males and 6 females) were used as a control group for the FSCV experiments, without any exposure or behavioral training. Experiments were carried out in accordance with the guidelines of the E.C. regulations for animal use in research (CEE no. 86/609) and our local ethics committee (CREMEAP; no. APAFIS#2145).

### 2.3 Anxiety-like behavior: Light-dark box test (LDB)

**FIGURE 1:**
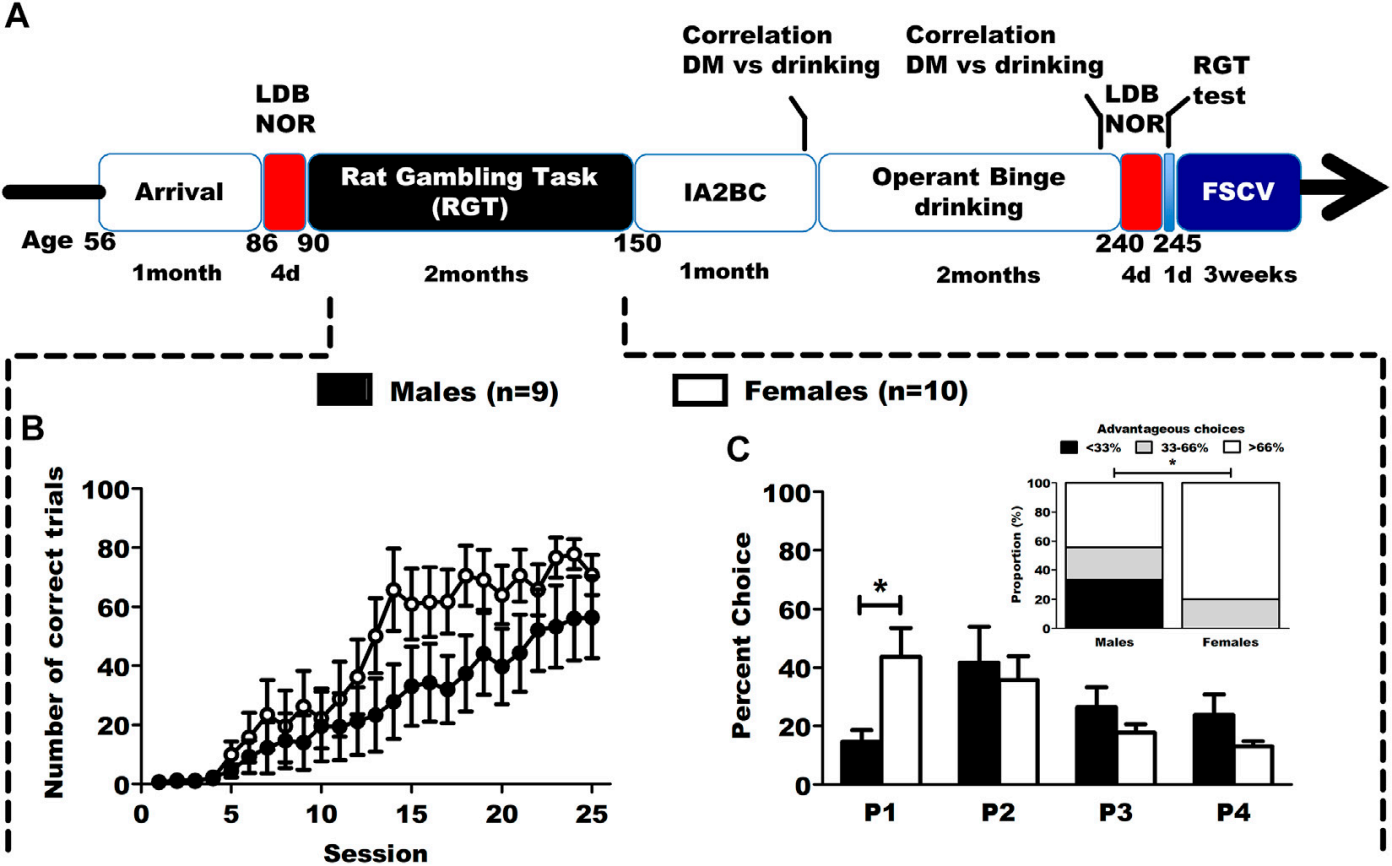
Timeline of the study and baseline results in the RGT. **(A)** Timeline of the longitudinal study. Animals were tested in DM capacities, anxiety (LDB) and learning (NOR) both before and after an operant binge drinking procedure. At the end of the behavioral experiments, the animals (as well as a control group without behavioral training) were sacrificed to assess mesolimbic phasic dopamine transmission in the core of the NAc, using *ex vivo* FSCV. **(B)** Correct trials during the first phase of RGT training. Results are expressed as mean ± SEM of the proportion of correct trials over the total number of trials during training sessions. **(C)** Choice behavior in the baseline session of RGT, and distribution of the DM level in categories (poor < 33%, neutral 33%–66% and good > 66%). The male rats favored the optimal P2 option, while the female rats favored both P1 and P2. The male group had significantly more individuals with a poor DM level than the female group. Results are expressed as mean ± SEM of the percent choice for each option, and as number of rats in each categories of decision makers. ^#^ *p* < 0.01, *p < 0.05.

Anxiety-like behavior was performed using the light dark box (LDB). The test was carried out before and after voluntary BD exposure (see Figure 1 timeline). Behavioral testing took place in a calm room inside two opaque plexiglass boxes (45 × 45 × 45 cm), divided into two equal compartments by a central partition (15 × 15 cm): an open lit compartment (30 lux), and a closed dark compartment (two Lux). The rats were habituated daily to the room for 1 hour during 1 week (5 consecutive days), inside their home cage, and the test sessions happened the day following the last habituation session. The animals were transferred to the room 30 min prior to every behavioral test. The test consisted in a single 5 min session where the animals were positioned in the lit compartment, back to the central door, and were allowed to freely move inside the box. All test sessions were performed during the same day, with an approximate time of 2 min between rats. The entry latency, the time spent inside the lit compartment and the number of transitions between compartments were recorded. A transition was recorded when all four limbs of the animal passed the central partition. The boxes were cleaned after each trial to prevent a bias based on olfactory cues.

### 2.4 Learning and memory: Novel object recognition task (NOR)

Learning abilities were assessed using the Novel Object Recognition test (NOR). The test was carried out before and after voluntary BD exposure (see Figure 1 timeline). Behavioral testing took place the day following the LDB test in the same calm room, inside two brightly illuminated (30 lux) opaque Plexiglas boxes (45 × 45 × 45 cm). The task consisted in three phases, one each consecutive day, for a total of 3 days: habituation, acquisition and test. The animals were transferred to the room 30 min prior to every behavioral test. The rats were first habituated to the boxes during a single 10 min session where they were allowed to move freely. During the acquisition phase, two objects (A and B) of distinct height, form and structure, were placed equidistant to the wall in each corner of the boxes. The rats were allowed to freely explore them for a single 10 min session. During the test phase, one object (counterbalanced for each box) was replaced with a new object (C) of different height, form and structure. The rats were allowed to once again freely explore them for a single 10 min session. There was an approximate time of 2 min between rats testing. Exploration time was recorded for each object, with any behavior of sniffing, licking or touching considered as interaction. The boxes and objects were cleaned after each trial to prevent a bias based on olfactory cues. A new set of different objects was used for the NOR test after ethanol exposure to limit retest effects.

### 2.5 Decision-making: Rat gambling task (RGT)

#### 2.5.1 Apparatus

Behavioral testing took place in four identical operant chambers (Imetronic, Pessac, France, 28 × 30 × 34 cm) in a calm room. Each chamber was standing in a dark, ventilated and sound-proof conditioning box. The chambers were divided into two equal compartments by a central plexiglass partition (0.5 × 29.5 × 30 cm), parallel to the wall and opened in its center (7 × 7 cm). They were also equipped on one side of the box with four nose-poke holes on a curved wall (dimly illuminated within with a white LED), equidistant from the food magazine at the opposite side. The nose-poke holes were equipped with an infrared detector connected to an external dispenser for the delivery of food pellets (45 mg, Test Diet, Cambridge Univ., United Kingdom). The apparatus and data collection were controlled using the POLY software (Imetronic, Pessac, France). The animals were moderately food restricted during the procedure (95% of their normal bodyweight).

DM was assessed using a rat gambling protocol inspired from the IGT in humans and developed by Zeeb et al. (2009), better suited for longitudinal studies than other available options [see (de Visser et al., 2011)]. The rats were alcohol naïve during the whole training phase of the RGT and the first test phase before the operant self-administration procedure (see Figure 1 timeline). Briefly, rats had to nose poke in four holes with different magnitude and probability of rewards and punishments. The rats were first habituated to the chamber during two 30 min sessions, during which food pellets were placed inside the nose-poke holes and the food magazine. During daily training, trials started with the animal visiting the food magazine, triggering the start of a 5 s Inter-Trial-Interval (ITI). Rats were first required to nose-poke into a briefly illuminated hole (0.5 s, randomly varying between each trial) within 10 s to earn a reward (one sugar pellet). A lack of response was recorded as an omission, while a response during the ITI was recorded as premature, both ending the ongoing trial and not giving a reward. Each session lasted for a maximum of 30 min and 100 trials. Training was pursued 5 days a week until a performance criterion of more than 80% correct trials and less than 20% omissions was achieved. To ensure equal experience with all future contingencies, the rats were then trained on a forced-choice version of the RGT for 7 sessions, where each nose-poke was associated with a different reward and punishment probability. The contingencies were such that the more rewarding options were associated with higher punishment in the form of timeouts (P1: 1 pellet *p* = 0.9, 5 s timeout *p* = 0.1; P2: 2 pellets *p* = 0.8, 10 s timeout *p* = 0.2; P3: 3 pellets *p* = 0.5, 30 s timeout *p* = 0.5; P4: 4 pellets *p* = 0.4, 6 0 s timeout *p* = 0.5; see Supplementary Table S1). One hole was illuminated per trial following a chronological order, and a rewarded trial consisted in the delivery of the pre-set number of pellets and signaled by onset of the tray light until collection of the food. Punishment consisted in a time-out window of the pre-set amount of time and signaled by the tray light remaining off and flashing of the stimulus light within the selected hole (frequency of 0.5 Hz). Training phase lasted between 3 weeks and more than a month.

#### 2.5.2 Test phase

Test phase consisted in daily 30 min free-choice RGT sessions. The trial design and their contingencies were the same, except that they started with the stimulus lights being turned on in all of the active holes. P1 and P2 options are considered as the advantageous choices, while P3 and P4 are considered as disadvantageous choices. If a rat chooses only one option, then the greatest number of pellets possible would be with P2 (411, most optimal option), then P1 (295), P3 (135) and P4 (99, least optimal option) (Zeeb et al., 2009). The animals were split in 2 groups receiving a different configuration of response outcomes in the holes (left-right counterbalance) to ensure no spatial or bias preference. All procedures were performed as previously described (Zeeb et al., 2009; Georgiou et al., 2018), with the exception of test sessions being limited (5 to 7 sessions) to the animals only displaying a stable preference for one of the options (3 consecutive sessions with the same option preferred), in order to avoid the development of an inflexible choice behavior. Here, we purposely limited the amount of test sessions, as it has been suggested that a prolonged training can lead to a highly robust choice behavior that is difficult to pharmacologically modulate (Spoelder et al., 2015). Reducing familiarity with the task also provide more face validity regarding the IGT, and is closer to a design supposed to involve both an exploratory and an exploitative phase (Buelow and Suhr, 2009).

### 2.6 Voluntary ethanol administration using an operant self-administration procedure

BD behavior was generated after the first RGT test (see Figure 1 timeline), with a protocol combining intermittent access to 20% ethanol in a two-bottle choice procedure (IA2BC) followed by an operant self-administration procedure in skinner cages. All the procedures have been previously described in one of our previous works (Jeanblanc et al., 2019). First, the rats had access to two bottles in their home cage, one containing tap water and the other a solution of 20% ethanol (v/v), every other day for 3 weeks. The bottles were placed and removed at 2:00 p.m., and weighed at the end of each drinking session. Two bottles were placed in an empty cage to control for the spillage of liquid. The rats were then trained daily to self-administer ethanol (0.1 mL of a 20% ethanol solution w/v per delivery) during the operant self-administration procedure. They were first submitted to two overnight sessions of 16 h (fixed ratio 1, FR1, between 5:00 p.m. and 9:00 a.m.) followed by shorter sessions 5 days a week (between 2:00 p.m. and 5:00 p.m.) with the following schedule: FR1—1 h for 5–7 days, FR3—1 h for 5–7 days and finally FR3—30 min until reaching a stable baseline of drinking (less than 20% of variation for three consecutive sessions. 8 days on average for males, 9 days on average for females). The sessions were then reduced to 15 min until leading to a stable BD phenotype with intoxicating levels of self-administration. Both male and female rats underwent 37 days of FR3-15 min sessions during this experiment. The number of active, inactive lever presses and rewards were recorded. Behavioral experiments were conducted 24 h after the end of the procedure.

### 2.7 Mesolimbic phasic dopamine transmission: Fast-scan cyclic voltammetry (FSCV)

#### 2.7.1 Surgery

Rats were anesthetized with isoflurane (IsoVet, 5%) before being decapitated, and their brain were extracted and immersed in ice-cold artificial cerebrospinal fluid (aCSF) (NaCl 126 mM, KCl 2.5 mM, NaH_2_PO_4_ 1.1 mM, MgCl_2_ 1.4 mM, CaCl_2_ 0.5 mM, NaHCO_3_ 18 mM, Glucose 11 mM, ascorbic acid 0.4 mM, pH 7.2–7.4) and glued into a vibratome (Leica, VT 1200S). Coronal slices (250 µm thick) of the NAc were selected and stored in a 31°C aCSF (NaCl 126 mM, KCl 2.5 mM, NaH_2_PO_4_ 1.1 mM, MgCl_2_ 1.4 mM, CaCl_2_ 2.4 mM, NaHCO_3_ 18 mM, Glucose 11 mM, pH 7.2–7.4) reservoir gassed with carbogen (95% O_2_, 5% CO_2_) for at least 1 hour. After rest, the slices were transferred to a recording chamber and perfused with aCSF (3 mL/min, 31°C).

#### 2.7.2 Recordings

DA measurements were conducted in the NAc core using FSCV and carbon-fiber microelectrodes (7 µm diameter, cut to 100–150 µm long, GoodFellow, Huntington, England). Those electrodes were calibrated prior to the recordings using a 1 µM dopamine solution in aCSF. A triangular waveform potential ramping from −0.4 V to +1.3 V was applied to the microelectrodes at 10 Hz during the recordings. A bipolar stimulating electrode (Stimulus Isolator A360, WPI, England) was placed on the afferent fibers coming from the VTA, around 100 µm near the working carbon-fiber microelectrode. Phasic DA release was evoked every 5 minutes using a monophasic stimulation (0.5 s long, 24 pulses, square-wave pulses, 2 m/phase, 300 μA, 60 Hz). The electric signal was amplified, filtered and transmitted to the Tarheel CV software (Scott Ng-Evans, Electronics and Materials Engineering Shop, Seattle, WA, United States). Using color plots, changes in current were plotted as a function of applied potential over time, and the oxidation current converted to DA concentration. A baseline of three consecutive stable signals was obtained for each slice. For the pharmacology, quinpirole 100 nM was applied for at least 30 min and until achieving three consecutive stable signals.

After performing the behavioral experiments (48 h to 1 week), rats were euthanized and coronal slices containing the NAc were collected to measure electrically evoked dopamine transmission in the core of the NAc. Rats from the control group (6 males and 6 females) were euthanized and their coronal slices collected 1 week after their arrival (57 weeks). The sensitivity to the D2/D3 receptor agonist quinpirole (100 nM) was tested.

#### 2.7.3 Data analysis

All analysis of release and uptake were conducted on the concentration-versus-time traces. These traces were fit to a model describing dopamine signaling as a balance between release and uptake, using the Michaelis-Menten equation (see, (Wu et al., 2001)), with the Lvit software (Scott Ng-Evans, Electronics and Materials Engineering Shop, Seattle, WA, United States). The equation is as follow:

$$\frac{d\left[DA\right]}{dt}=f{\left[DA\right]}_{p}-\frac{Vmax}{\left(\frac{Km}{\left[DA\right]}\right)+1}$$

where [*DA*] is the instant extracellular concentration of *DA* released, *f* is the frequency of stimulation, [DA]_p_ is dopamine concentration released per pulse, and Vmax and Km are respectively the velocity and affinity constants of the dopamine transporter (DAT). Km was fixed at a constant value of 200 nM (Wu et al., 2001). [DA]_p_ is the reflect of the presynaptic mechanisms regulating release as those of the auto-receptors (D2/D3 activity) (Kennedy et al., 1992). Peak dopamine concentration was extracted for each trace before fitting to the model in order to obtain a [DA]_max_ value, reflecting maximum extracellular dopamine concentration, and was used as a parameter of dopamine release. [DA]_p_ and V_max_ values were modulated until fitting the traces to the model, with a correlation coefficient of 0.8 or more with our experimental data, using the Lvit software.

### 2.8 Statistical analysis

Data were analyzed using SigmaPlot 11.0 (SysStat Software, Inc.) with an analysis of variance test (2-way ANOVA or 1-way ANOVA, with repeated measures when appropriate) followed by a Tukey multiple comparison test, when a significant effect was observed for the data following a normal distribution and an equality of variances. When the data did not follow these criteria, a non-parametric analysis was performed (Friedman’s test or Kruskal-Wallis test and Dunn’s *post hoc* test). For single comparison, we used a Student’s t-test (2-tailed) for the data following a normal distribution and an equality of variances, and a Mann-Whitney rank sum test otherwise. For proportions, we used a *χ* ^2^ test. For the correlation analysis, we used a Pearson correlation test. All data were presented as Means ± standard error means (SEM). The significance threshold was fixed at *p* < 0.05.

## 3 Results

A time line of the study is presented on Figure 1.

All the statistical analyses are provided in Supplementary Table S2.

### 3.1 Sex differences observed in baseline training and testing in the RGT

The animals underwent 8 weeks of daily training in the RGT, and had to learn to nose-poke in the operant holes to earn a reward. During the first 25 sessions of training (Figure 1B; Supplementary Table S1), females rats seemed to increase their percentage of correct trials quicker than males although no statistically significant differences were found (2way-RM ANOVA: session F_(24,191)_ = 20.306, *p* < 0.001; sex F_(1,191)_ = 2.899, *p* = 0.127; interaction F_(24,191)_ = 1.180, *p* = 0.264). Thereafter, we evaluated their baseline choice behavior in the RGT on the last 3 sessions. Male rats favored the most advantageous option (P2) and female rats favored both advantageous options (P1 and P2) (Figure 1C; Supplementary Table S1, 2way-RM ANOVA: sex F_(1,24)_ = 0.346, *p* = 0.573; option F_(3,24)_ = 0.1436, *p* = 0.257; interaction F_(3,24)_ = 5.740, *p* = 0.004). Female rats chose the P1 option significantly more than male rats (Tukey, *p* < 0.001). Distribution of poor (<33% good choices), neutral (≥33–≤66%) and good (>66% good choices) decision makers was different depending on sex (Figure 1C, *χ* ^2^_(2)_ = 6.14, *p* < 0.05). Regarding the other RGT parameters, no sex differences were observed for advantageous choices, number of trials and premature responses, but there were more omitted trials in females (Supplementary Figure S1).

### 3.2 BD impaired DM in males but not females

**FIGURE 2:**
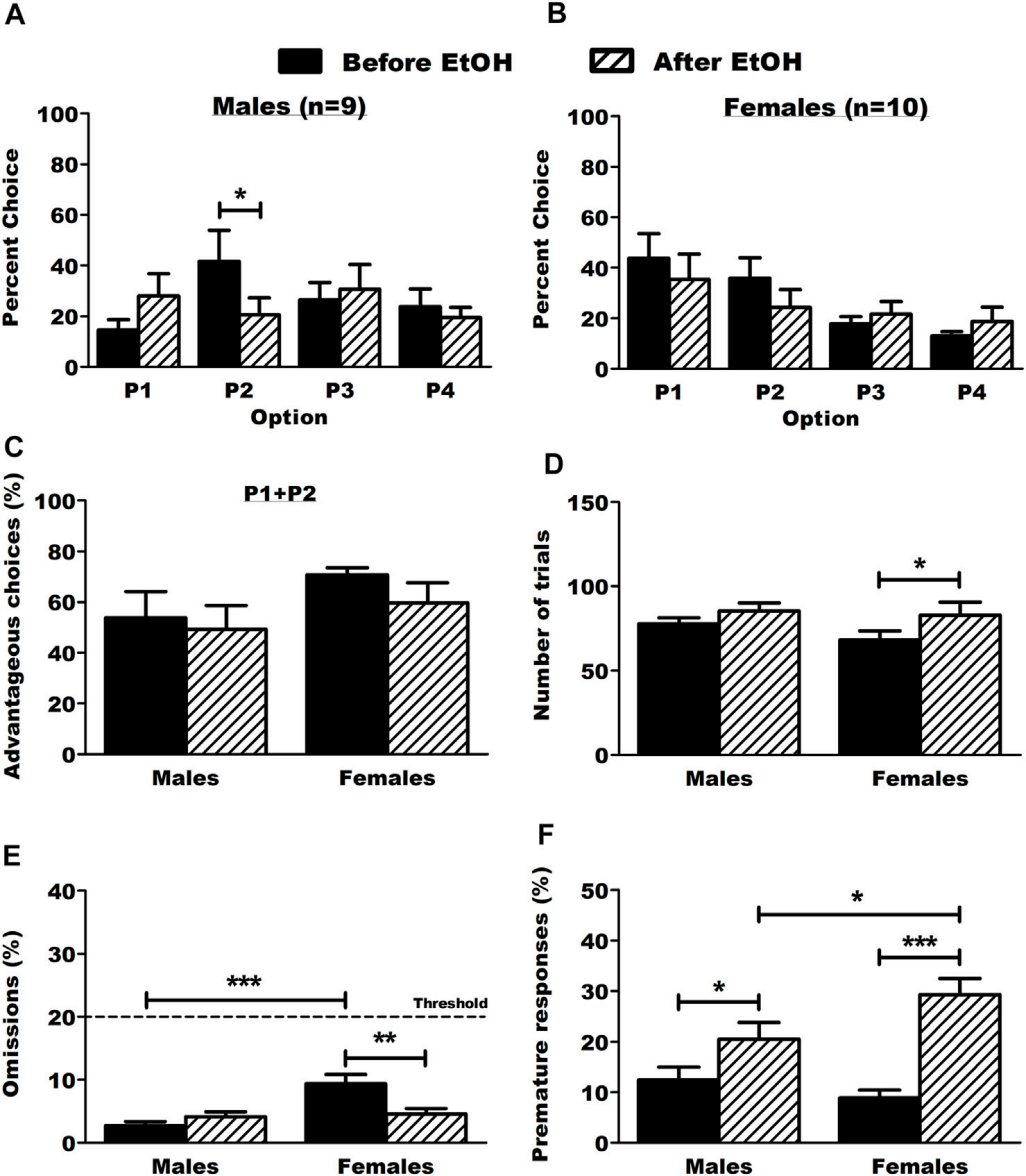
Choice behavior and other parameters in the RGT before and after ethanol. **(A)** Male rats chose the optimal option P2 significantly less after ethanol. Results are expressed as mean ± SEM of the percent choice of each option. **(B)** Ethanol did not affect choice behavior in female rats. Results are expressed as mean ± SEM of the percent choice of each option. **(C)** Ethanol did not affect the proportion of advantageous choices (P1 + P2) in both males and female rats. Results are expressed as mean ± SEM of the proportion of advantageous choices over the total number of choices. **(D)** Ethanol increased the number of trials in female rats. Results are expressed as mean ± SEM of the total number of trials made. **(E)** Ethanol decreased omissions in female rats. The dot line represents the threshold of allowed omitted responses for the animals during baseline test sessions. The male rats omitted more trials than the female rats before ethanol. Results are expressed as mean ± SEM of the proportion of omitted trials over the total number of trials. **(F)** Ethanol increased the proportion of premature responses in both male and female rats. The female rats made more premature responses than the male rats after ethanol. Results are expressed as mean ± SEM of the proportion of premature responses over the total number of trials. *p < 0.05, **p < 0.01, ***p < 0.001.

After the self-administration procedure, one male rat was excluded from the group due to sickness. Results from the operant BD procedure are detailed in Supplementary Figure S2. Briefly, Escalation in both ethanol intake and preference was observed in both sexes and there were no sex differences in ethanol intake during the operant self-administration, thus ruling out the potential bias of differential ethanol intake during the 3 months of BD history. Although we did not assess blood ethanol concentrations in our rats, we expect mean concentrations between 40 and 50 mg/dL, according to our previous work showing an almost linear positive correlation between ethanol intake in the BD procedure and blood ethanol concentrations (Jeanblanc et al., 2018). The RGT test was done 24 h after the last operant session (BD sessions were maintained in between behavioral experiments). The BD history changed DM capacities with a decrease in P2 choices specifically in males (Figure 2A, 2way RM-ANOVA: treatment F_(1,18)_ = 26.761, *p* < 0.001; option F_(3,18)_ = 0.390, *p* = 0.761; interaction F_(3,18)_ = 2.694, *p* = 0.069; Tukey *p* < 0.05) but not in females (Figure 2B, 2way RM-ANOVA: treatment F_(1,24)_ = 3.006, *p* = 0.117; option F_(3,24)_ = 2.363, *p* = 0.093; interaction F_(3,24)_ = 1.314, *p* = 0.290). There were no effects on advantageous choices (P1+P2, Figure 2C, 2way RM-ANOVA: sex F_(1,6)_ = 1.902, *p* = 0.186; treatment F_(1,6)_ = 2.170, *p* = 0.159; interaction F_(1,6)_ = 0.380, *p* = 0.546) and only females displayed a small increase in their number of trials (Figure 2D, 2way RM-ANOVA: sex F_(1,6)_ = 0.725, *p* = 0.406; treatment F_(1,6)_ = 6.484, *p* = 0.021; interaction F_(1,6)_ = 0.643, *p* = 0.434; Tukey *p* < 0.05). Females made significantly more omissions than male rats before BD onset and they reduced their number of omissions after a voluntary BD history (Figure 2E, 2way RM-ANOVA: sex F_(1,6)_ = 9.378, *p* = 0.007; treatment F_(1,6)_ = 3.722, *p* = 0.071; interaction F_(1,6)_ = 12.657, *p* = 0.002; Tukey *p* < 0.001 vs. males, *p* < 0.01 vs. after EtOH). Both males and females made significantly more premature responses after voluntary BD history and this effect was more pronounced in females (Figure 2F, 2way RM-ANOVA: sex F_(1,6)_ = 0.824, *p* = 0.377; treatment F_(1,6)_ = 30.164, *p* < 0.001; interaction F_(1,6)_ = 5.697, *p* = 0.029; Tukey *p* < 0.05 males vs. females after EtOH, *p* < 0.05 in males before EtOH vs. after EtOH, *p* < 0.001 in females before EtOH vs. after EtOH). An additional analysis including the sex factor for choice behavior in each option of the RGT is provided in the supplementary material (Supplementary Figure S3).

### 3.3 Correlations between DM abilities at baseline, ethanol intake and FSCV parameters

**FIGURE 3:**
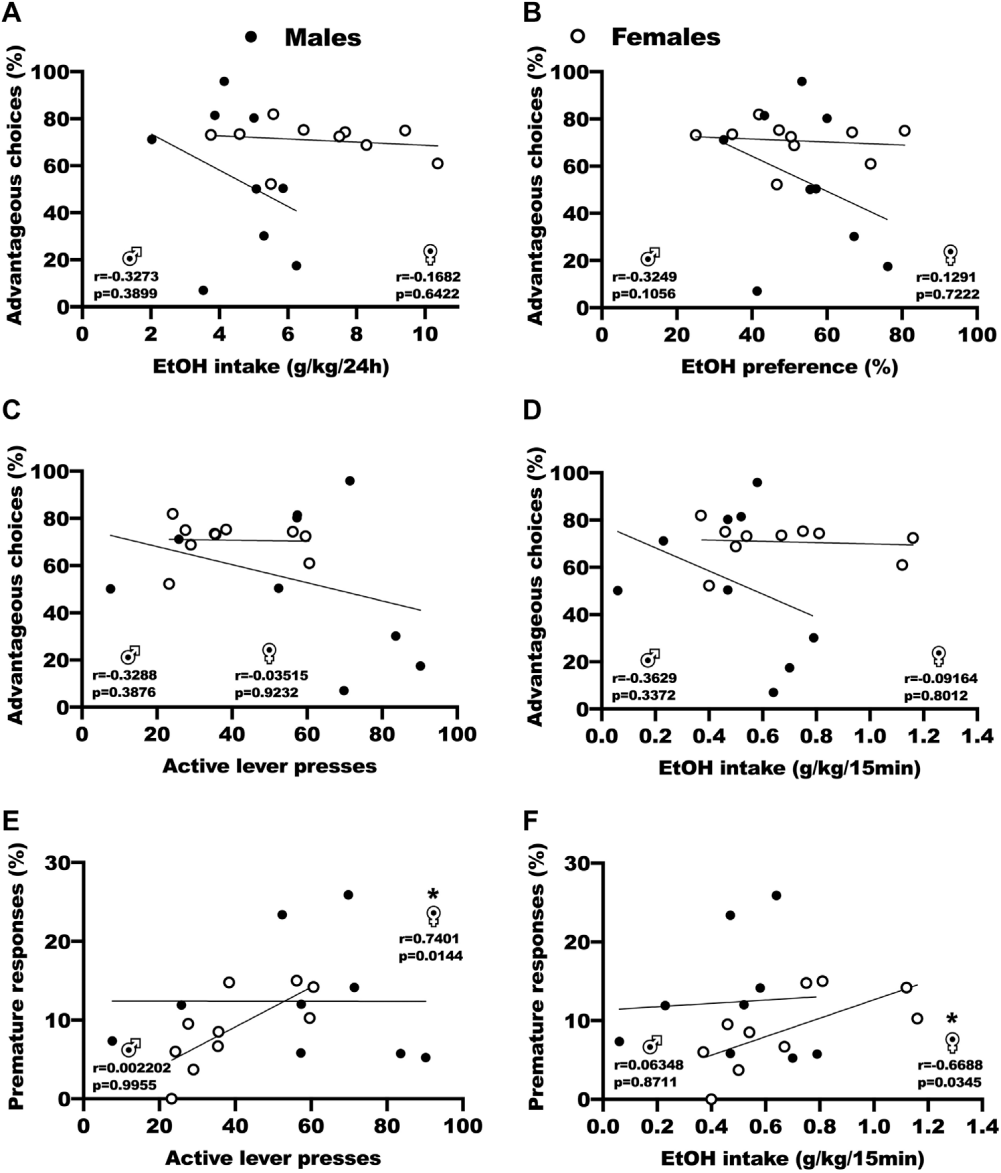
Correlation analysis of the relation between baseline DM abilities and ethanol consumption. **(A)** No correlation between advantageous choices (P1 + P2) during baseline RGT sessions and ethanol intake by weight during the last IA2BC session. Results are expressed as the percent of choices in the P1 and P2 options versus the ethanol intake by weight during the 24 h sessions (g/kg/24 h). **(B)** No correlation between advantageous choices (P1 + P2) during baseline RGT sessions and ethanol preference during the last IA2BC session. Results are expressed as the percent of choices in the P1 and P2 options versus the proportion of ethanol consumed over total fluid consumed. **(C)** No correlation between advantageous choices (P1 + P2) during baseline RGT sessions and active lever presses during the last 5 stable operant self-administration FR3 15 min sessions. Results are expressed as the percent of choices in the P1 and P2 options versus the number of presses on the active lever during the 15 min sessions. **(D)** No correlation between advantageous choices (P1 + P2) during baseline RGT sessions and ethanol intake by weight during the last 5 stable operant self-administration FR3 15 min sessions. Results are expressed as the percent of choices in the P1 and P2 options versus the ethanol intake by weight during the 15 min sessions (g/kg/15 min). **(E)** Positive correlation in females, but not males, between premature responses during baseline RGT sessions and the number of presses on the active lever during the last 5 stable operant self-administration FR3 15 min sessions. Results are expressed as the percent of premature responses versus the number of presses on the active lever during the 15 min sessions. **(F)** Positive correlation in females, but not males, between premature responses during baseline RGT sessions and ethanol intake by weight during the last 5 stable operant self-administration FR3 15 min sessions. Results are expressed as the percent of premature responses versus the ethanol intake by weight during the 15 min sessions (g/kg/15 min). *p < 0.05.

We used correlation analysis to assess the relation between baseline DM performances in the RGT, the following voluntary ethanol consumption in the IA2BC and operant self-administration procedures, and the FSCV parameters (Figure 3; Supplementary Table S3). For both sexes, Pearson correlation tests revealed no significant correlation between advantageous choices (P1+P2) during baseline RGT test sessions and ethanol intake by weight during the last session of IA2BC (Figure 3A, r = −0.33 and *p* = 0.389 for males, r = −0.17 and *p* = 0.642 for females), ethanol preference during the last session of IA2BC (Figure 3B, r = 0.32 and *p* = 0.106 for males, r = 0.13 and *p* = 0.722 for females), active lever presses during the last 5 stable FR3 15 min operant self-administration sessions (Figure 3C, r = −0.33 and *p* = 0.388 for males, r = −0.01 and 0.923 for females), and ethanol intake during the last 5 stable FR3 15 min operant self-administration sessions (Figure 3D, r = 0.36 and *p* = 0.337 for males, r = −0.09 and *p* = 0.801 for females). Pearson correlation tests however revealed a positive correlation in female rats between premature responses and active lever presses (Figure 3E, r = 0.7401 and *p* = 0.144)/ethanol intake (Figure 3F, r = −0.6688 and *p* = 0.0345) during the last 5 stable FR3 15 min operant self-administration sessions, but not in male rats (r = −0.3288 and *p* = 0.3876 for active lever presses, r = −0.3629 and *p* = 0.3372 for ethanol intake). Outside of the ones we are showing in this manuscript, we found no correlation between the other parameters from the RGT, the BD procedure and the FSCV (Supplementary Table S3).

### 3.4 BD history has no effects on memory and anxiety-like behavior

#### 3.4.1 Learning and memory

**FIGURE 4:**
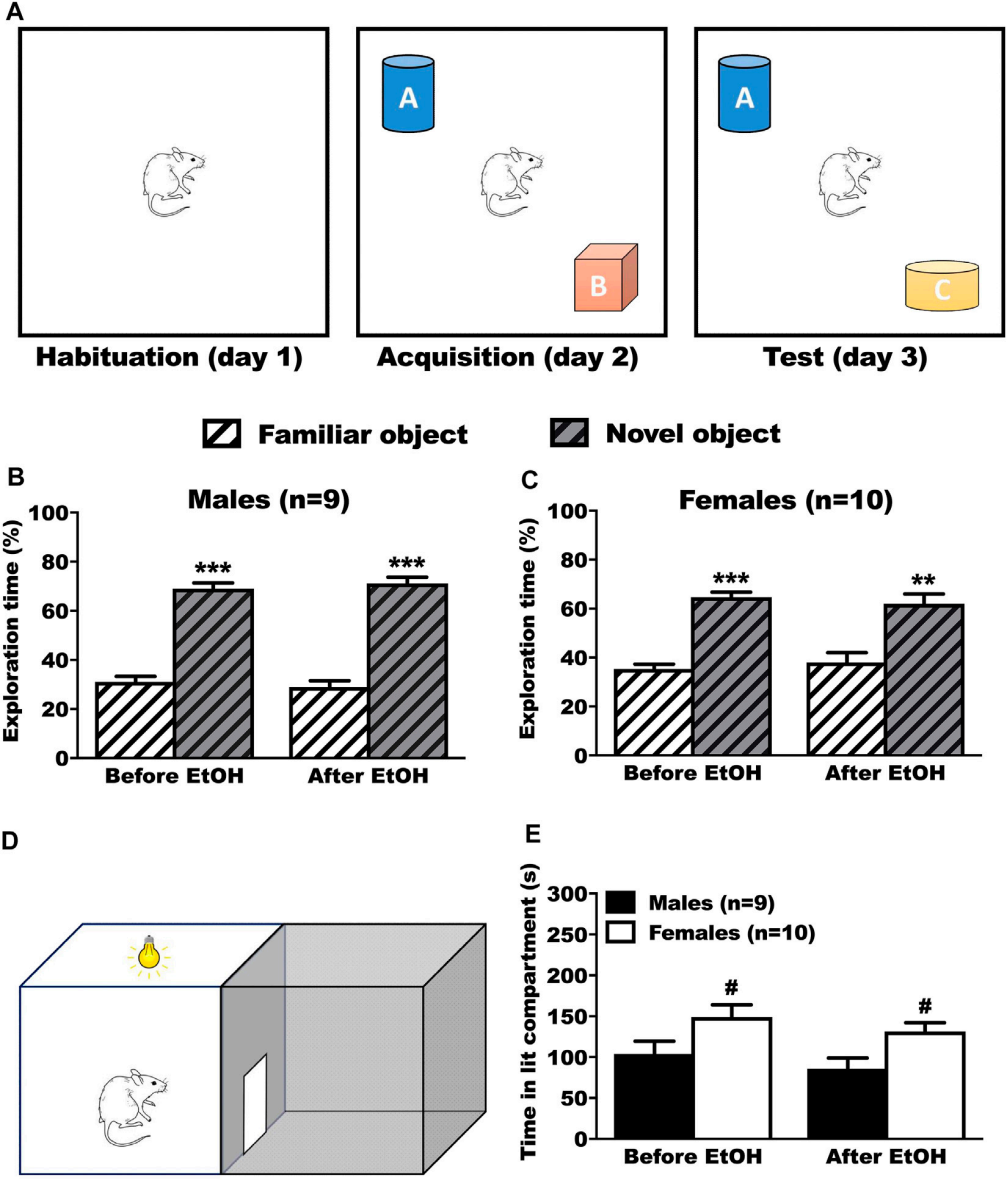
Results of the anxiety (LDB) and learning/memory test (NOR) before and after ethanol. **(A)** Timeline of the NOR test. After exploring the box during the habituation phase, animals had the chance to explore 2 different objects (A and B) or during the acquisition phase. During the test phase, one of the objects was switched with a new different one (Novel Object, C), while the other remained (Familiar Object, A). **(B,C)** No differences in performances were observed before and after ethanol. Male **(B)** and female **(C)** rats spent significantly more time on the novel object, showing that they memorized the familiar object, both before and after ethanol. Results are expressed as mean ± SEM of the proportion of time spent exploring each object over the total time spent exploring. **(D)** Schematic representation of the DLB test. The animal was positioned in the lit compartment, back to the central door, and allowed to freely move inside the box. **(E)** No effect of ethanol on the time spent inside the lit compartment during the LDB test. Female rats spent significantly more time inside the lit compartment than the male rats, both before and after ethanol. Results are expressed as mean ± SEM of the time in seconds spent inside the lit compartment during the test. *p < 0.05, **p < 0.01, ***p < 0.001 vs. familiar object. ^#^ *p* < 0.05 vs. males.

In the NOR test, the rats were presented with 2 objects and tested with retention in presence of a new object again 24 h later (Figure 4A). We evaluated the time spent on the familiar and the novel object, before and after voluntary ethanol. The male rats explored the novel object significantly more than the familiar object before (Tukey *p* < 0.001) and after (Tukey *p* < 0.001) BD (Figure 4B, 2way RM-ANOVA: object F_(1,34)_ = 211.05, *p* < 0.001; treatment F_(1,34)_ = 0.01, *p* = 1.000; interaction F_(1,34)_ = 0.20, *p* = 0.655). The female rats explored the novel object significantly more than the familiar object, both before (Tukey *p* < 0.001) and after (Tukey *p* = 0.002) BD (Figure 4C, 2way RM-ANOVA: object F_(1,9)_ = 38.12, *p* < 0.001; treatment F_(1,9)_ = 0.01, *p* = 1.000; interaction F_(1,9)_ = 0.35, *p* = 0.569).

#### 3.4.2 Anxiety

In the DLB test (Figure 4D), the rats were left for 5 min to explore the box. We evaluated the time spent in the lit compartment. The male rats spent significantly less time in the lit compartment than female rats, both before (Tukey *p* = 0.021) and after (Tukey *p* = 0.02) BD (Figure 4E, 2way RM-ANOVA: sex F_(1,17)_ = 8.38, *p* = 0.010; treatment F_(1,17)_ = 3.38, *p* = 0.084, interaction F_(1,17)_ = 0.01, *p* = 0.988).

### 3.5 Effects of BD history on baseline phasic dopamine signaling 48 h after the last ethanol exposure

**FIGURE 5:**
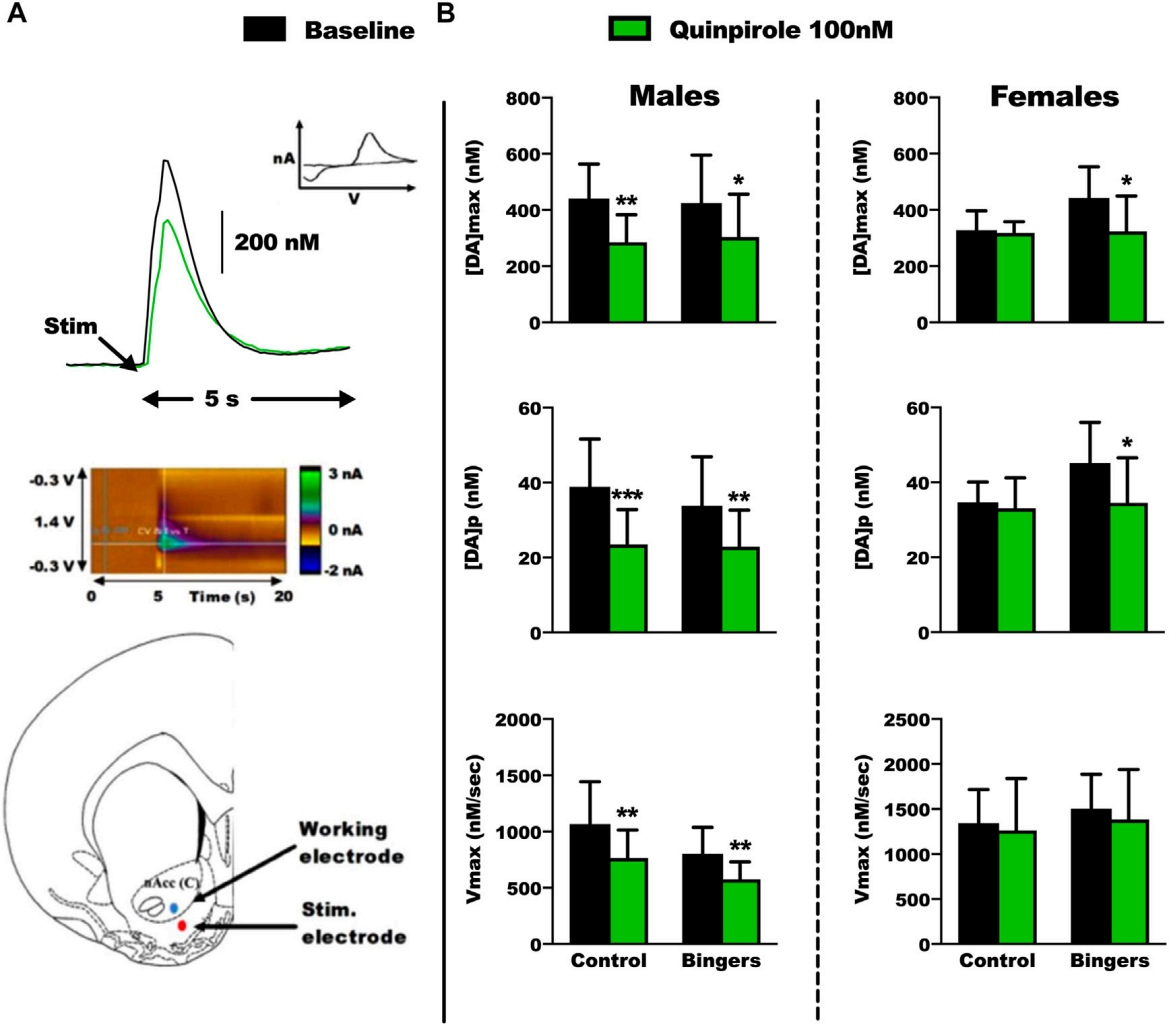
*Ex-vivo* FSCV results in male and female rats. Control: *n* = 6 males and *n* = 6 females, Bingers: *n* = 8 males and *n* = 7 females. **(A)** Top panel: Example of a FSCV trace from a control male rat, and characteristic voltamogramm of dopamine. Middle panel: Example of color plot from a control male rat during the FSCV recordings. Bottom panel: Electrode placements for the FSCV recordings in the core of the NAc. **(B)** Top panel: results for [DA]_max_ in male and female rats. Results are expressed as mean ± SEM of the maximum extracellular [DA], in nM. Middle panel: results for [DA]_p_ in male and female rats. Results are expressed as mean ± SEM of the [DA] per pulse of stimulation, in nM. Bottom panel: results for V_max_ in male and female rats. Results are expressed as mean ± SEM of the dopamine transporter velocity, in nM/sec. *p < 0.05, **p < 0.01, ***p < 0.001 vs. respective baseline.

We evaluated the phasic dopamine signaling induced by electrical stimulation in the core of the NAc using FSCV on brain slices, in both male and female rats, after behavioral experiments. Thus, both DM capacities and dopaminergic signaling were assessed in the same animals. We tested a 100 nM dose of quinpirole on the slices to unravel potential changes in the sensitivity of the D2/3 receptors (Figure 5; Supplementary Table S2 for all statistical analyses).

At baseline, we did not observe any changes in the dopamine signaling parameters between control and rats with an history of BD. In general, and as expected, quinpirole decreased dopamine signaling and all parameters of the different groups of males. Strikingly, this effect is lacking in the control group of females but observed after a history of exposure to BD.

In males, quinpirole decreased [DA]max in control (Tukey *p* = 0.002) and binger rats (Tukey *p* = 0.036) (Figure 5B top left panel, 2way RM-ANOVA: treatment F_(2,10)_ = 24.715, *p* < 0.001; group F_(1,10)_ = 0.00594, *p* = 0.442; interaction F_(2,10)_ = 1.007, *p* = 0.399) [DA]p in control (Tukey *p* < 0.001) and binger rats (Tukey *p* = 0.002) (Figure 5B middle left panel, 2way RM-ANOVA: treatment F_(2,10)_ = 33.906, *p* < 0.001; group F_(1,10)_ = 0.116, *p* = 0.747; interaction F_(2,10)_ = 1.328, *p* = 0.308), and Vmax in control (Tukey *p* = 0.002) and binger rats (Tukey *p* = 0.009) (Figure 5B bottom left panel, 2way RM-ANOVA: treatment F_(2,10)_ = 12.283 *p* = 0.002; group F_(1,10)_ = 3.787, *p* = 0.109; interaction F_(2,10)_ = 1.156, *p* = 0.354).

In females, quinpirole decreased [DA]max in binger (Tukey *p* = 0.01) but not control rats (Figure 5B top right panel, 2way RM-ANOVA: treatment F_(2,10)_ = 8.231, *p* = 0.008; group F_(1,10)_ = 0.605, *p* = 0.472; interaction F_(2,10)_ = 1.780, *p* = 0.218), [DA]p in binger (Tukey *p* = 0.011) but not control rats (Figure 5B middle right panel, 2way RM-ANOVA: treatment F_(2,10)_ = 10.346 *p* = 0.004; group F_(1,10)_ = 1.828, *p* = 0.234; interaction F_(2,10)_ = 1.387 *p* = 0.294), and had no effect on Vmax (Figure 5B bottom right panel, 2way RM-ANOVA: treatment F_(2,10)_ = 1.394 *p* = 0.292; group F_(1,10)_ = 0.949, *p* = 0.375; interaction F_(2,10)_ = 0.0214, *p* = 0.979).

## 4 Discussion

The present study outlines that a chronic voluntary BD exposure impairs DM capacities specifically in male rats. Those DM impairments were not associated with an increase in cognitive deficits or in anxiety-like behavior. They were also not associated with impairments in dopaminergic transmission in the core of the NAc. In females, despite no effects on choice behavior, BD history altered impulsive control and increased sensitivity to the inhibitory effect of quinpirole on dopamine release thus suggesting adaptation in D2/3 receptor functioning.

Sex differences in DM capacities were seen before the onset of BD. Although we found no significant statistical differences, it seemed like most females were faster in acquiring the task which may be explained by better attention skills, despite making more omissions. As expected, sex differences were obvious regarding preferences for options: i) no poor decision makers in females; ii) males significantly preferred the optimal P2 option and iii) females preferred both P1 and P2 options. The P1 option, preferred by females, can be seen as a “risk-averse” phenotype, as it is associated with the lowest probability of punishment, as previously suggested (Georgiou et al., 2018). This observation is in line with human studies in which women use a different choice strategy than men, displaying more sensitivity to punishment frequency and occasional losses in the IGT (van den Bos et al., 2013).

Voluntary BD impaired choice behavior specifically in males, with a decrease of the optimal P2 option choice (50% decrease), although without effects on the overall advantageous choices made. BD history in males made their choice behavior become “random” (around 25% choice for each option), as they could not effectively select the advantageous options in the task anymore. Our results are in line with those showing impaired DM in conflictual and risky situations after both forced (Nasrallah et al., 2011; Boutros et al., 2015) and voluntary (McMurray et al., 2014; 2016) binge-like procedures in male rodents. Clinical studies have also shown impairment of DM abilities in adolescent bingers (Goudriaan et al., 2007; 2011; Johnson et al., 2008). Male rats did not significantly increase the less advantageous choices (more immediate reward with more punishment), suggesting that they did not display a state or trait of hypersensitivity to reward or increased risk-taking as previously suggested in humans (Johnson et al., 2008). Instead, rats were unable to discern options for their value and adapt from feedback, as described in alcohol addiction (for review, (Verdejo-Garcia et al., 2018)), but not yet in the case of BD. A previous preclinical study using low dose i.p. ethanol during the acquisition of the task found similar results (Spoelder et al., 2015). Our results show that DM impairments were not linked to changes in anxiety-like behavior in the LDB or cognitive deficits in the NOR. BD also notably increased motor impulsivity in both sexes (increased premature responses) in line with heightened motor impulsivity previously described in humans (Sanchez-Roige et al., 2014). It is also important to note that the second RGT measurements were performed during acute ethanol withdrawal, but we did not notice any acute withdrawal symptoms that may have affected behavior in the task.

One of the most striking result of our study is that BD history had no effect on DM capacities in female rats. Many human studies have shown that women are more sensitive to the effects of alcohol than men, but sex differences regarding BD remain poorly investigated (for review, (Wilsnack et al., 2017)). Some studies have found worse performances in global executive functioning and DM in male bingers (Parada et al., 2012), while others have found similar impairments in both sexes (Goudriaan et al., 2007; Carbia et al., 2017). In the present study, it is noteworthy that before BD onset, the female group displayed more good decision makers than the male group, and no poor decision makers. In addition, the males also displayed a higher level of anxiety than females, a trait that has already been associated with poor DM (Miu et al., 2008). Those results could suggest that good baseline DM performances are less sensitive to the effects of alcohol as shown in well-trained animals (Spoelder et al., 2015). In agreement with this observation, we showed in a previous study (Jeanblanc et al., 2019) using a different RGT procedure [as described in (Rivalan et al., 2009)], that alcohol could preferentially impair individuals with neutral or poor performances, while not affecting those with good performances. On the contrary, individuals with poor DM abilities could be more vulnerable. For instance, it has been shown that rats with poor DM abilities in the RGT display extreme scores in risk taking, reward seeking, behavioral inflexibility and motor impulsivity (Rivalan et al., 2013). Working on female rats also raises the question of the role of the hormonal cycle. However, our females were housed individually in ventilated racks and therefore did not synchronize their hormonal cycle. As such, there is little risk of a statistically significant effect of the phase of the cycle on our results. Moreover, we have previously confirmed in our laboratory that the hormonal cycle does not affect ethanol consumption in our BD procedure (data not shown), and previous studies have shown that it has no effect on the IGT in humans (van den Bos et al., 2013), and the RGT (Georgiou et al., 2018) in rats.

No study has yet analyzed whether DM abilities can predict the vulnerability to consume ethanol in individuals who have never used ethanol before. The use of a longitudinal design allowed us to assess the vulnerability to consume ethanol in the BD procedure, but the analysis revealed no correlation between baseline DM abilities (advantageous choices: P1 + P2) and ethanol consumption in the IA2BC and operant self-administration procedures (Figure 3). Interestingly, we found a positive correlation in females (but not males) between premature responses and active lever presses/ethanol intake during the FR3 15 min self-administration sessions. As premature responses in the task reflect motor impulsivity, this could mean that female animals with a high base level of impulsivity could end up drinking more in our task. We did not find any correlation between the other parameters in the RGT, the BD procedure and the FSCV data (Supplementary Table S3). It is however important to note that our sample size was not optimal for such analysis, and would probably require more animals. Thus, our results overall do not support the widespread intuition that poor DM abilities may increase vulnerability to drink ethanol, but rather that chronic ethanol intake is responsible for DM impairments in a sex specific manner. Some results in the literature indicate that cognitive deviations and personality traits (impulsivity, sensation seeking, risk taking…) accompanying addiction, rather than drug consumption in itself, may explain DM impairments (Kovács et al., 2017). The relation between drug consumption and preexisting impulsivity, risk taking or DM impairments has however been shown in a very mild and inconstant manner for alcohol and other drugs, with a remaining uncertain causality (Ahmed, 2018).

Our results on dopamine transmission demonstrate that BD does not affect basal dopamine signaling in the NAc core, regardless of sex, but reveal sex-dependent changes in the sensitivity to the inhibitory effects of quinpirole. Baseline dopamine signaling (release and uptake) was similar in both sexes in control groups. Our results are in line with those of a recent meta-analysis on 39 microdialysis studies showing that there are no sex-dependent differences in basal dopamine levels within the NAc (Egenrieder et al., 2020). It is noteworthy that other studies have however suggested sex-dependent differences in dopamine signaling (Walker et al., 1999; Walker et al., 2006). Thus, our results on basal dopamine signaling cannot explain our behavioral results showing sex-differences in baseline training and testing in the RGT, as we initially hypothesized. Using a bigger sample size, it would however be interesting to compare baseline dopamine signaling between different DM groups. In a previous work, we indeed found a significant difference in DA release between male rats with good and poor DM levels (although using a different RGT protocol and having a possible confounding effect of ethanol) (Jeanblanc et al., 2019).

Baseline dopamine signaling was not altered in both sexes after a history of BD exposure. Our result is in line with those of a recent study that used a forced BD exposure, although in adolescent male rats, and showed no changes in baseline dopamine signaling using *in vivo* FSCV at adulthood (Shnitko et al., 2016). To the best of our knowledge, no studies have analyzed the effects of a BD protocol on striatal dopaminergic signaling using *ex vivo* FSCV. It may be that basal phasic dopamine signaling is only impaired using protocols to induce alcohol dependence, but not BD (Karkhanis et al., 2015). While quinpirole reduced dopaminergic signaling in males and the history of BD had no effect, in females, we found that the history of BD may have induced neuro-adaptations making the dopaminergic transmission (release but not the transporter since the V_max_ is unchanged) sensitive to the inhibitory effect of quinpirole. It seems that autoreceptors are less sensitive to quinpirole in females with no BD history. A previous study using FSCV in female rhesus macaques showed that 1 year of daily ethanol self-administration induced greater dopamine uptake rates and sensitivity to D2 autoreceptors in the core of the NAc, thus driving a hypodopaminergic state (Siciliano et al., 2016). Altogether, these results suggest sex differences in striatal dopamine signaling and that alcohol exposure history may also differently affect release mechanisms depending upon sex-related factors. Previous findings indicate that dopamine neurotransmission is differently regulated in male and female rats (Walker et al., 2006; Georgiou et al., 2018). Contrary to what we would expect from our results, preclinical studies supporting sex differences in D2 receptors expression show that females have a higher D2 density in the striatum (Williams et al., 2021). Age could also be a factor in our results, especially the lack of effect of our quinpirole dose in female controls, as our animals were far into adulthood and it has been shown that D2 receptor density is declining in the striatum, although seemingly with a greater exponential decline in males than females (Williams et al., 2021). Finally, it is important to note that the fact that our control group did not undergo any behavioral procedure could influence our dopamine recordings and the responses to pharmacology. Phasic DA release in the NAc is involved with associative learning during operant conditioning (Roitman et al., 2004; Owesson-White et al., 2008), and active administration of drugs is associated with neuroadaptations in specific cognitive processes (Jacobs et al., 2003).

It is interesting to note that our behavioural results cannot be clearly explained by our FSCV data. As only male rats showed DM impairments with BD, we expected to observe phasic signaling impairments specifically in them, but only female rats were affected by BD in their sensitivity to quinpirole. It seems like impairments in DM processes are not explained by modifications in basal dopamine signaling or pre-synaptic D2 activity. The effects of D2 modulation on DM using the same RGT protocol than we did remains inconclusive. It has been shown that the selective D2 dopamine receptor antagonist eticlopride increased advantageous choices specifically in males, while quinpirole increased advantageous choices specifically in females (Zeeb et al., 2009). Other studies have found no effect at all on DM (Di Ciano et al., 2015) or only with a concomitant serotoninergic modulation (Di Ciano et al., 2018). Although the NAc core is important in value-based reward, being limited to it in *ex vivo* FSCV may explain the lack of a causality link between behavioral and neurobiological data. It will be important to use whole brain setups such as *in vivo* FSCV in the future to better understand how DM processes are affected by chronic ethanol.

## 5 Conclusion

Overall, our study emphasize that BD exposure impairs both DM processes and dopamine signaling in the core of the NAc in a sex-related manner, further suggesting that these effects may play a role in the vicious cycle leading to BD perpetuation and the early onset of AUD and dependence. We further advocate for the use of our operant model of BD, which shows better face validity and leads to changes in behavior without negative effects (behavioral suppression, inflammation, stress…) compared to classic passive exposures (injection, gavage…). So far, clinical results on the sex differences in the BD field of research looking at brain and cognitive deficits are still inconclusive and need more investigation. Thus, the overall difference in BD exposure raises the important question about the BD history that is also often matter of debate in human studies because it is difficult to have homogeneous population of binge drinkers with the same BD history.

## Data Availability

The original contributions presented in the study are included in the article/Supplementary Materials, further inquiries can be directed to the corresponding author: MN, mickael.naassila@u-picardie.fr.

## References

[B1] Adan A. Forero D. A. Navarro J. F. (2017). Personality traits related to binge drinking: A systematic review. Front. Psychiatry 8, 134–211. 10.3389/fpsyt.2017.00134 28804465 PMC5532381

[B2] Ahmed S. H. (2018). Individual decision-making in the causal pathway to addiction: Contributions and limitations of rodent models. Pharmacol. Biochem. Behav. 164, 22–31. 10.1016/j.pbb.2017.07.005 28709784

[B3] Ashenhurst J. R. Harden K. P. Corbin W. R. Fromme K. (2016). Trajectories of binge drinking and personality change across emerging adulthood. Psychol. Addict. Behav. 29 (4), 978–991. 10.1037/adb0000116 PMC4701625 26348219

[B4] Becker J. B. Hu M. (2008). Sex differences in drug abuse. Front. Neuroendocrinol. 29, 36–47. 10.1016/j.yfrne.2007.07.003 17904621 PMC2235192

[B5] Bø R. Billieux J. Landrø N. I. (2016). Binge drinking is characterized by decisions favoring positive and discounting negative consequences. Addict. Res. Theory 24 (6), 499–506. 10.3109/16066359.2016.1174215

[B6] Boutros N. (2015). Adolescent intermittent ethanol exposure is associated with increased risky choice and decreased dopaminergic and cholinergic neuron markers in adult rats, 1–9. 10.1093/ijnp/pyu003 PMC4368879 25612895

[B7] Budygin E. A. Phillips P. E. Wightman R. M. Jones S. R. (2001b). Terminal effects of ethanol on dopamine dynamics in rat nucleus accumbens: An *in vitro* voltammetric study. Synapse 42 (2), 77–79. 10.1002/syn.1101 11574942

[B8] Budygin E. A. Phillips P. E. Robinson D. L. Kennedy A. P. Gainetdinov R. R. Wightman R. M. (2001a). Effect of acute ethanol on striatal dopamine neurotransmission in ambulatory rats. J. Pharmacol. Exp. Ther. 297, 27–34. 11259524

[B9] Buelow M. T. Suhr J. A. (2009). Construct validity of the Iowa gambling task. Neuropsychol. Rev. 19 (1), 102–114. 10.1007/s11065-009-9083-4 19194801

[B10] Cappelli C. Ames S. Shono Y. Dust M. Stacy A. (2017). Affective decision-making moderates the effects of automatic associations on alcohol use among drug offenders. Am. J. Drug Alcohol Abuse 43 (5), 534–544. 10.1080/00952990.2016.1216557 27624979 PMC6097230

[B11] Carbia C. Cadaveira F. Caamano-Isorna F. Rodriguez Holguin S. Corral M. (2017). Binge drinking trajectory and decision-making during late adolescence: Gender and developmental differences. Front. Psychol. 8, 783–810. 10.3389/fpsyg.2017.00783 28555122 PMC5430068

[B12] de Visser L. Homberg J. R. Mitsogiannis M. Zeeb F. D. Rivalan M. Fitoussi A. (2011). Rodent versions of the Iowa gambling task: Opportunities and challenges for the understanding of decision-making. Front. Neurosci. 5 (OCT), 109–121. 10.3389/fnins.2011.00109 22013406 PMC3189637

[B13] Di Chiara G. Imperato A. (1988). Drugs abused by humans preferentially increase synaptic dopamine concentrations in the mesolimbic system of freely moving rats. Proc. Natl. Acad. Sci. U. S. A. 85 (14), 5274–5278. 10.1073/pnas.85.14.5274 2899326 PMC281732

[B14] Di Ciano P. Manvich D. F. Pushparaj A. Gappasov A. Hess E. J. Weinshenker D. (2018). Effects of disulfiram on choice behavior in a rodent gambling task: Association with catecholamine levels. Psychopharmacology 235, 23–35. 10.1007/s00213-017-4744-0 29085979 PMC5750121

[B15] Di Ciano P. Pushparaj A. Kim A. Hatch J. Masood T. Ramzi A. (2015). The impact of selective dopamine D2, D3 and D4 ligands on the rat gambling task. PLoS ONE 10 (9), 01362677–e136316. 10.1371/journal.pone.0136267 PMC4564230 26352802

[B16] Di Ciano P. Robbins T. W. Everitt B. J. (2008). Differential effects of nucleus accumbens core, shell, or dorsal striatal inactivations on the persistence, reacquisition, or reinstatement of responding for a drug-paired conditioned reinforcer. Neuropsychopharmacology 33 (6), 1413–1425. 10.1038/sj.npp.1301522 17712353

[B17] Doumas D. M. Miller R. Esp S. (2017). Impulsive sensation seeking, binge drinking, and alcohol-related consequences: Do protective behavioral strategies help high risk adolescents. Addict. Behav. 64, 6–12. 10.1016/j.addbeh.2016.08.003 27533076 PMC10662253

[B18] Dreyer J. K. Vander Weele C. M. Lovic V. Aragona B. J. (2016). Functionally distinct dopamine signals in nucleus accumbens core and shell in the freely moving rat. J. Neurosci. 36 (1), 98–112. 10.1523/jneurosci.2326-15.2016 26740653 PMC6601791

[B19] Egenrieder L. Mitricheva E. Spanagel R. Noori H. R. (2020). No basal or drug‐induced sex differences in striatal dopaminergic levels: A cluster and meta‐analysis of rat microdialysis studies. J. Neurochem. 152 (4), 482–492. 10.1111/jnc.14911 31705667

[B20] Ernst M. Paulus M. P. (2005). Neurobiology of decision making: A selective review from a neurocognitive and clinical perspective. Biol. Psychiatry 58 (8), 597–604. 10.1016/j.biopsych.2005.06.004 16095567

[B21] Evans K. L. Hampson E. (2015). Sex differences on prefrontally-dependent cognitive tasks. Brain Cognition 93, 42–53. 10.1016/j.bandc.2014.11.006 25528435

[B22] Georgiou P. Zanos P. Bhat S. Tracy J. K. Merchenthaler I. J. McCarthy M. M. (2018). Dopamine and stress system modulation of sex differences in decision making’, *Neuropsychopharmacology* . Nat. Publ. Group 43 (2), 313–324. 10.1038/npp.2017.161 PMC5729565 28741626

[B23] Gierski F. Benzerouk F. De Wever E. Duka T. Kaladjian A. Quaglino V. (2017). Cloninger's temperament and character dimensions of personality and binge drinking among college students. Students 41 (11), 1970–1979. 10.1111/acer.13497 28902418

[B24] Goudriaan A. E. Grekin E. R. Sher K. J. (2007). Decision making and binge drinking: A longitudinal study. A Longitud. Study 31 (6), 928–938. 10.1111/j.1530-0277.2007.00378.x PMC2667377 17403069

[B25] Goudriaan A. E. Grekin E. R. Sher K. J. (2011). Decision making and response inhibition as predictors of heavy alcohol use: A prospective study. Alcohol Clin. Exp. Res. 35 (6), 1050–1057. 10.1111/j.1530-0277.2011.01437.x 21332527 PMC3097267

[B26] Hooper C. J. Luciana M. Conklin H. M. Yarger R. S. (2004). Adolescents’ performance on the Iowa gambling task: Implications for the development of decision making and ventromedial prefrontal cortex. Dev. Psychol. 40, 1148–1158. 10.1037/0012-1649.40.6.1148 15535763

[B27] Imperato A. Di Chiara G. (1986). Preferential stimulation of dopamine release in the nucleus accumbens of freely moving rats by ethanol. J. Pharmacol. Exp. Ther. 239 (239), 219–228. 3761194

[B28] Jacobs E. H. Smit A. B. de Vries T. J. Schoffelmeer A. N. M. (2003). Neuroadaptive effects of active versus passive drug administration in addiction research. Trends Pharmacol. Sci. 24 (11), 566–573. 10.1016/j.tips.2003.09.006 14607079

[B29] Jeanblanc J. Rolland B. Gierski F. Martinetti M. P. Naassila M. (2018). Animal models of binge drinking, current challenges to improve face validity. Neurosci. Biobehav. Rev. 68, 112–121. 10.1016/j.neubiorev.2018.05.002 29738795

[B30] Jeanblanc J. Sauton P. Jeanblanc V. Legastelois R. Echeverry-Alzate V. Lebourgeois S. (2019). Face validity of a pre-clinical model of operant binge drinking: Just a question of speed. Addict. Biol. 24 (4), 664–675. 10.1111/adb.12631 29863763

[B31] Johnson C. A. Xiao L. Palmer P. Sun P. Wang Q. Wei Y. (2008). Affective decision-making deficits, linked to a dysfunctional ventromedial prefrontal cortex, revealed in 10th grade Chinese adolescent binge drinkers. Reveal. 10th grade Chin. Adolesc. binge drinkers 46, 714–726. 10.1016/j.neuropsychologia.2007.09.012 PMC3498846 17996909

[B32] Jones S. R. Mathews T. A. Budygin E. A. (2006). Effect of moderate ethanol dose on dopamine uptake in rat nucleus accumbens *in vivo* . Synapse 60 (3), 251–255. 10.1002/syn.20294 16752364

[B33] Karkhanis A. N. Rose J. H. Huggins K. N. Konstantopoulos J. K. Jones S. R. (2015). Chronic intermittent ethanol exposure reduces presynaptic dopamine neurotransmission in the mouse nucleus accumbens. Drug Alcohol Dependence 150, 24–30. 10.1016/j.drugalcdep.2015.01.019 25765483 PMC4387104

[B34] Kennedy R. T. Jones S. R. Wightman R. M. (1992). Dynamic observation of dopamine autoreceptor effects in rat striatal slices. J. Neurochem. 59 (2), 449–455. 10.1111/j.1471-4159.1992.tb09391.x 1352798

[B35] Kovács I. Richman M. J. Janka Z. Maraz A. Ando B. (2017). Decision making measured by the Iowa gambling task in alcohol use disorder and gambling disorder: A systematic review and meta-analysis. Drug Alcohol Dependence 181, 152–161. 10.1016/j.drugalcdep.2017.09.023 29055269

[B36] Kuntsche E. Kuntsche S. Thrul J. Gmel G. (2017). Binge drinking: Health impact, prevalence, correlates and interventions. Psychol. Health 32, 976–1017. 10.1080/08870446.2017.1325889 28513195

[B37] Lannoy S. Billieux J. Dormal V. Maurage P. (2019). Behavioral and cerebral impairments associated with binge drinking in youth: A critical review. Psychol. Belg. 59 (1), 116–155. 10.5334/pb.476 31328014 PMC6625552

[B38] Leeman R. F. Hoff R. A. Krishnan-Sarin S. Patock-Peckham J. A. Potenza M. N. (2015). Impulsivity, sensation-seeking and part-time job status in relation to substance use and gambling in adolescents. J. Adolesc. Health 54 (4), 460–466. 10.1016/j.jadohealth.2013.09.014 PMC4048016 24268362

[B39] Mathews T. A. John C. E. Lapa G. B. Budygin E. A. Jones S. R. (2006). No role of the dopamine transporter in acute ethanol effects on striatal dopamine dynamics. Synapse 60, 288–294. 10.1002/syn.20301 16786536

[B40] McFarland K. Lapish C. C. Kalivas P. W. (2003). Prefrontal glutamate release into the core of the nucleus accumbens mediates cocaine-induced reinstatement of drug-seeking behavior. J. Neurosci. 23 (8), 3531–3537. 10.1523/jneurosci.23-08-03531.2003 12716962 PMC6742291

[B41] McMurray M. S. Amodeo L. R. Roitman J. D. (2016). Consequences of adolescent ethanol consumption on risk preference and orbitofrontal cortex encoding of reward. Neuropsychopharmacology 41, 1366–1375. 10.1038/npp.2015.288 26370327 PMC4793121

[B42] McMurray M. S. Amodeo L. R. Roitman J. D. (2014). Effects of voluntary alcohol intake on risk preference and behavioral flexibility during rat adolescence. PLoS ONE 41 (5), e100697–e101375. 10.1371/journal.pone.0100697 PMC4090063 25007338

[B43] Miu A. C. Heilman R. M. Houser D. (2008). Anxiety impairs decision-making: Psychophysiological evidence from an Iowa gambling task. Biol. Psychol. 77, 353–358. 10.1016/j.biopsycho.2007.11.010 18191013

[B44] Nasrallah N. A. Clark J. J. Collins A. L. Akers C. A. Phillips P. E. Bernstein I. L. (2011). Risk preference following adolescent alcohol use is associated with corrupted encoding of costs but not rewards by mesolimbic dopamine. Proc. Natl. Acad. Sci. 108 (13), 5466–5471. 10.1073/pnas.1017732108 21402915 PMC3069180

[B45] Owesson-White C. A. Cheer J. F. Beyene M. Carelli R. M. Wightman R. M. (2008). Dynamic changes in accumbens dopamine correlate with learning during intracranial self-stimulation. Proc. Natl. Acad. Sci. U. S. A. 105 (33), 11957–11962. 10.1073/pnas.0803896105 18689678 PMC2575325

[B46] Parada M. Corral M. Mota N. Crego A. Rodriguez Holguin S. Cadaveira F. (2012). Executive functioning and alcohol binge drinking in University students’, *Addictive Behaviors* . Elsevier Ltd. 37 (2), 167–172. 10.1016/j.addbeh.2011.09.015 21996093

[B47] Pascual M. Boix J. Felipo V. Guerri C. (2009). Repeated alcohol administration during adolescence causes changes in the mesolimbic dopaminergic and glutamatergic systems and promotes alcohol intake in the adult rat. J. Neurochem. 108 (108), 920–931. 10.1111/j.1471-4159.2008.05835.x 19077056

[B48] Pelkonen A. Hiltunen M. Kiianmaa K. Yavich L. (2010). Stimulated dopamine overflow and alpha-synuclein expression in the nucleus accumbens core distinguish rats bred for differential ethanol preference. J. Neurochem. 114, 1168–1176. 10.1111/j.1471-4159.2010.06844.x 20533994

[B49] Philpot R. M. Wecker L. Kirstein C. L. (2009). Repeated ethanol exposure during adolescence alters the developmental trajectory of dopaminergic output from the nucleus accumbens septi. Int. J. Dev. Neurosci. 27 (8), 805–815. 10.1016/j.ijdevneu.2009.08.009 19712739

[B50] Rivalan M. Ahmed S. H. Dellu-Hagedorn F. (2009). Risk-prone individuals prefer the wrong options on a rat version of the Iowa Gambling Task’, *Biological Psychiatry* . Elsevier Inc. 66 (8), 743–749. 10.1016/j.biopsych.2009.04.008 19482266

[B51] Rivalan M. Valton V. Series P. Marchand A. R. Dellu-Hagedorn F. (2013). Elucidating poor decision-making in a rat gambling task. PLoS ONE 8 (12), e82052. 10.1371/journal.pone.0082052 24339988 PMC3855331

[B52] Robinson D. L. Howard E. C. McConnell S. Gonzales R. A. Wightman R. M. (2009). Disparity between tonic and phasic ethanol-induced dopamine increases in the nucleus accumbens of rats. Alcohol 33 (7), 1187–1196. 10.1111/j.1530-0277.2009.00942.x PMC2947861 19389195

[B53] Robinson D. L. Volz T. J. Schenk J. O. Wightman R. M. (2005). Acute ethanol decreases dopamine transporter velocity in rat striatum: *In vivo* and *in vitro* electrochemical measurements. Electrochem. Measurements’ 29 (5), 746–755. 10.1097/01.ALC.0000164362.21484.14 15897718

[B54] Roitman M. F. Stuber G. D. Phillips P. E. M. Wightman R. M. Carelli R. M. (2004). Dopamine operates as a subsecond modulator of food seeking. J. Neurosci. 24 (6), 1265–1271. 10.1523/JNEUROSCI.3823-03.2004 14960596 PMC6730321

[B55] Rolland B. Naassila M. (2017). Binge drinking: Current diagnostic and therapeutic issues. CNS Drugs 31 (3), 181–186. 10.1007/s40263-017-0413-4 28205146

[B56] Sanchez-Roige S. Baro V. Trick L. Pena-Oliver Y. Stephens D. N. Duka T. (2014). Exaggerated waiting impulsivity associated with human binge drinking, and high alcohol consumption in mice. Neuropsychopharmacol. 39, 2919–2927. 10.1038/npp.2014.151 PMC4229569 24947901

[B57] Shnitko T. A. Spear L. P. Robinson D. L. (2016). Adolescent binge-like alcohol alters sensitivity to acute alcohol effects on dopamine release in the nucleus accumbens of adult rats. Psychopharmacology 233 (3), 361–371. 10.1007/s00213-015-4106-8 26487039 PMC4840100

[B58] Siciliano C. A. Calipari E. S. Yorgason J. T. Lovinger D. M. Mateo Y. Jimenez V. A. (2016). Increased presynaptic regulation of dopamine neurotransmission in the nucleus accumbens core following chronic ethanol self-administration in female macaques. Psychopharmacology 233 (8), 1435–1443. 10.1007/s00213-016-4239-4 26892380 PMC4814331

[B59] Spanagel R. (2009). Alcoholism: A systems approach from molecular physiology to addictive behavior. Physiol. Rev. 89 (2), 649–705. 10.1152/physrev.00013.2008 19342616

[B60] Spoelder M. Lesscher H. M. B. Hesseling P. Baars A. M. Lozeman-van t Klooster J. G. Mijnsbergen R. (2015). Altered performance in a rat gambling task after acute and repeated alcohol exposure. Psychopharmacology 232 (19), 3649–3662. 10.1007/s00213-015-4020-0 26220611 PMC4561076

[B61] Stopper C. M. Floresco S. B. (2015). Dopaminergic circuitry and risk/reward decision making: Implications for schizophrenia. Schizophr. Bull. 41 (1), 9–14. 10.1093/schbul/sbu165 25406370 PMC4266315

[B62] Sugam J. A. (2012). Phasic nucleus accumbens dopamine encodes risk-based decision-making behavior. Nano 6 (9), 2166–2171. 10.1021/nl061786n.Core-Shell PMC3253943 22055017

[B63] Tavolacci M. P. Berthon Q. Cerasuolo D. Dechelotte P. Ladner J. Baguet A. (2019). Does binge drinking between the age of 18 and 25 years predict alcohol dependence in adulthood? A retrospective case-control study in France. BMJ Open 9 (5), e026375. 10.1136/bmjopen-2018-026375 PMC6501952 31061035

[B64] Trifilieff P. Martinez D. (2014). Imaging addiction: D2 receptors and dopamine signaling in the striatum as biomarkers for impulsivity. Neuropharmacology 76, 498–509. PART B). 10.1016/j.neuropharm.2013.06.031 23851257 PMC4106030

[B65] van den Bos R. Homberg J. de Visser L. (2013). A critical review of sex differences in decision-making tasks: Focus on the Iowa Gambling Task. Behav. Brain Res. 238 (1), 95–108. 10.1016/j.bbr.2012.10.002 23078950

[B66] Verdejo-Garcia A. Chong T. T. J. Stout J. C. Yucel M. London E. D. (2018). Stages of dysfunctional decision-making in addiction’, *Pharmacology Biochemistry and Behavior* . Failure:Problem retrieving ISSN S0091305716302817 164, 99–105. 10.1016/j.pbb.2017.02.003 28216068

[B67] Walker Q. D. Ray R. Kuhn C. M. (2006). Sex differences in neurochemical effects of dopaminergic drugs in rat striatum. Neuropsychopharmacology 31 (6), 1193–1202. 10.1038/sj.npp.1300915 16237396

[B68] Walker Q. D. Rooney M. B. Wightman R. M. Kuhn C. M. (1999). Dopamine release and uptake are greater in female than male rat striatum as measured by fast cyclic voltammetry. Neuroscience 95 (4), 1061–1070. 10.1016/S0306-4522(99)00500-X 10682713

[B69] Williams O. O. F. Coppolino M. George S. R. Perreault M. L. (2021). Sex differences in dopamine receptors and relevance to neuropsychiatric disorders. Brain Sci. 11 (9), 1199. 10.3390/brainsci11091199 34573220 PMC8469878

[B70] Wilsnack R. W. (2017). Gender differences in binge drinking prevalence, predictors, and consequences. Alcohol Res. Curr. Rev. 39 (1), e1–e20. 10.35946/arcr.v39.1.09 PMC6104960 30557149

[B71] Wu Q. Reith M. E. Wightman R. M. Kawagoe K. T. Garris P. A. (2001). Determination of release and uptake parameters from electrically evoked dopamine dynamics measured by real-time voltammetry. J. Neurosci. Methods 112 (2), 119–133. 10.1016/S0165-0270(01)00459-9 11716947

[B72] Yoo J. Y. Kim M. S. (2016). Deficits in Decision-Making and reversal learning in college students who participate in Binge drinking. Neuropsychiatry 6 (6), 1000156. 10.4172/Neuropsychiatry.1000156

[B73] Zack M. Poulos C. X. (2007). A D2 antagonist enhances the rewarding and priming effects of a gambling episode in pathological gamblers. Neuropsychopharmacology 32, 1678–1686. 10.1038/sj.npp.1301295 17203013

[B74] Zandy S. L. Matthews D. B. Tokunaga S. Miller A. Blaha C. D. Mittleman G. (2016). Reduced dopamine release in the nucleus accumbens core of adult rats following adolescent binge alcohol exposure: Age and dose-dependent analysis. Psychopharmacology 232 (4), 777–784. 10.1007/s00213-014-3712-1 PMC4351806 25116483

[B75] Zeeb F. D. Robbins T. W. Winstanley C. A. (2009). Serotonergic and dopaminergic modulation of gambling behavior as assessed using a novel rat gambling task. Nat. Publ. Group 34 (10), 2329–2343. 10.1038/npp.2009.62 19536111

