# Supplementary figures and images for: Sex-specific decision-making impairments and striatal dopaminergic changes after binge drinking history in rats
Source: Front Pharmacol. 2023 Jan 16;14:1076465. doi: 10.3389/fphar.2023.1076465 (PMC9885167; doi:10.3389/fphar.2023.1076465)

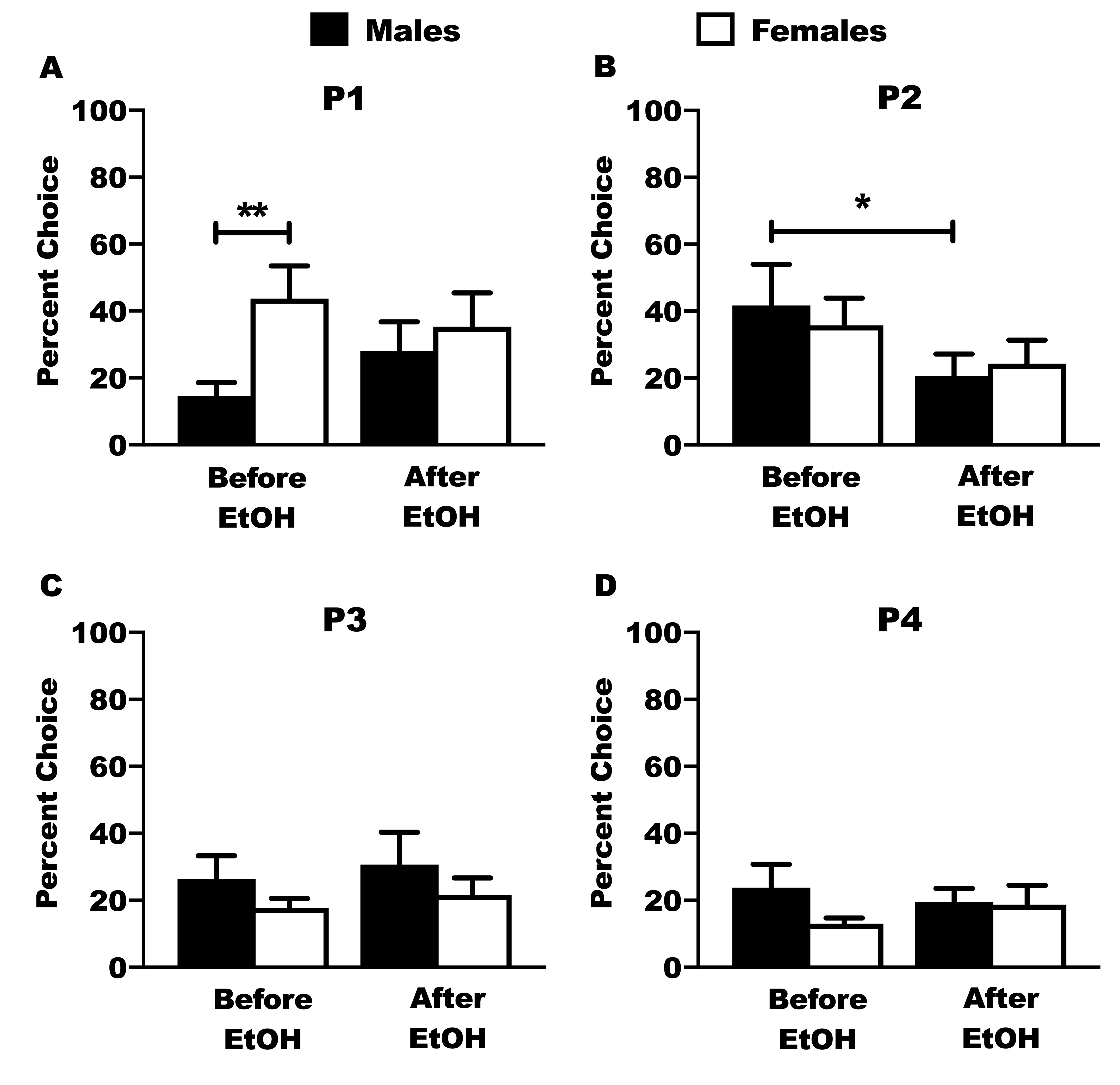

Supplement: Supplementary file 1 [file Image3.JPEG]

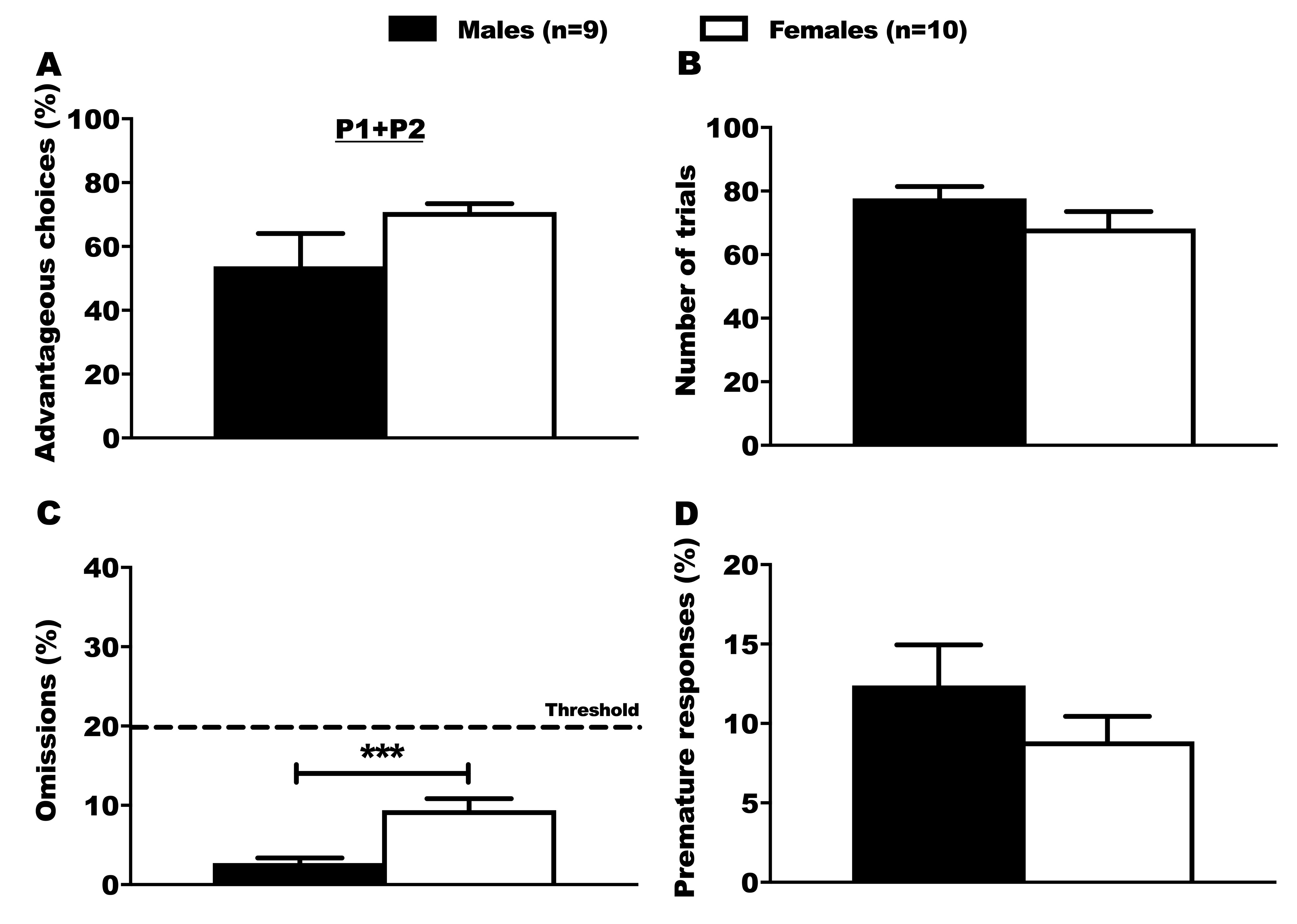

Supplement: Supplementary file 2 [file Image1.JPEG]

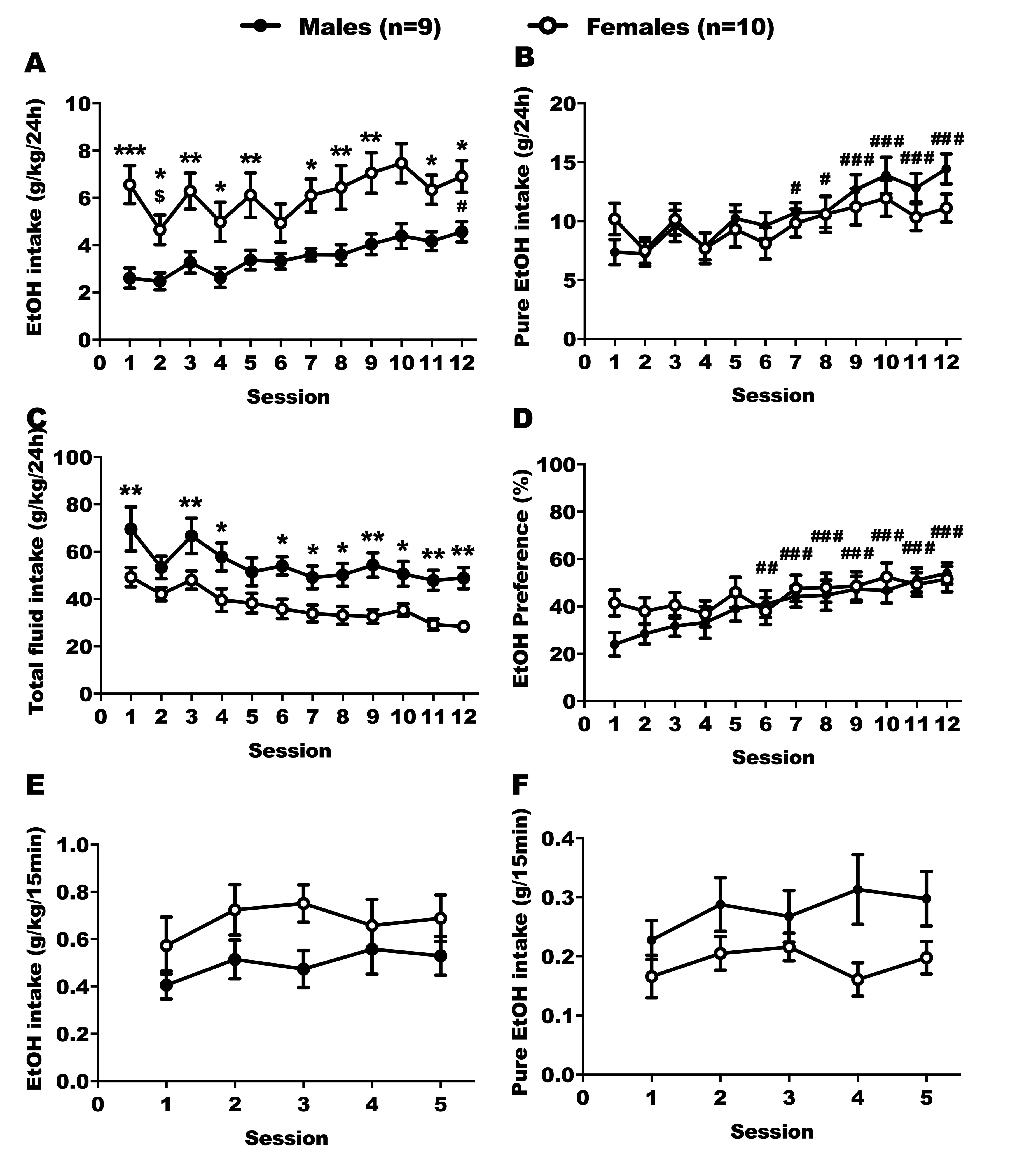

Supplement: Supplementary file 3 [file Image2.JPEG]
