# Supplementary material for: Sex-specific decision-making impairments and striatal dopaminergic changes after binge drinking history in rats
Source: Front Pharmacol. 2023 Jan 16;14:1076465. doi: 10.3389/fphar.2023.1076465 (PMC9885167; doi:10.3389/fphar.2023.1076465)
Supplement: Supplementary file 4 [file DataSheet1.docx]

Supplementary Material

# Supplementary Data

## Other parameters during baseline RGT test sessions

The RGT allows to evaluate the proportion of advantageous choices, as well as the number of trials, the proportion of omissions and the proportion of premature responses. We evaluated and compared these parameters between the male and female rats. Statistical analysis using Student’s t-test revealed marginally more premature responses (**Supplemental Figure 1D**, p=0.0830) in male rats than female rats, but no significant differences in the number of trials (**Supplemental Figure 1B**, p=0.1081). Statistical analysis using Mann-Whitney rank sum test revealed significantly less omissions (Supplemental Figure 1C, p=0.002) in the male rats than the female rats, but no significant differences in the percent of advantageous choices (**Supplemental Figure 1A**, p=0.804).

## Sex differences in the BD operant procedure

The operant BD procedure started with 12 sessions of the IA2BC paradigm, in order to develop ethanol palatability in the animals and escalate their ethanol intake. We first evaluated their ethanol intake relative to weight across the sessions. Female rats had a significantly higher ethanol intake relative to weight across sessions than male rats (Tukey p<0.001), but they didn’t escalate their intake relative to weight, while the male rats did by the end of the procedure (Tukey p=0.033 for session 12 vs the first) (**Supplemental Figure 2A**, 2way RM-ANOVA: sex F_(1,187)_=12.55, p=0.003; session F_(11,187)_=6.93, p<0.001; interaction (F_(11,187)_=1.13, p=0.343). We also evaluated pure intake of ethanol, showing that male rats significantly escalated their pure ethanol intake starting from the 7^th^ session (Tukey p=0.02 for session 7, p=0.014 for session 8 and p<0.001 for sessions 9 to 12) (**Supplemental Figure 2B**, 2way RM-ANOVA: sex F_(1,187)_=0.35, p=0.563; session F_(11,187)_=13.606, p<0.001; interaction F_(1,187)_=2.29, p=0.012). Then, we evaluated the total fluid intake, showing that male rats had a significantly higher total fluid intake than female rats (Tukey p=0.005 for session 1, p=0.009 for session 2, p=0.011 for sessions 4, p=0.011 for session 5, p=0.031 for session 7, p=0.017 for session 8, p=0.003 for session 9, p=0.031 for session 10, p=0.009 for session 11 and p=0.005 for session 12) (**Supplemental Figure 2C**, 2way RM-ANOVA: sex F_(1,187)_=11.03, p=0.004; session F_(11,187)_=8.04, p<0.001; interaction F_(11,187)_=0.44, p=0.934). We also evaluated the ethanol preference, with males significantly escalating their ethanol preference starting from the 6th session (Tukey p=0.007 for session 6, p<0.001 for sessions 7 to 12), but not female rats (**Supplemental Figure 3D**, 2way RM-ANOVA: sex F_(1,187)_=0.26, p=0.616; session F_(11,187)_=8.876, p<0.001; interaction F_(11,187)_=1.93, p=0.038). Finally, the rats underwent daily self-administration sessions for several weeks. We evaluated their ethanol intake and found no differences between sexes relative to ethanol intake by weight (**Supplemental Figure 2E**, 2way RM-ANOVA: sex F_(1,63)_=2.15, p=0.162; session F_(4,63)_=2.08, p=0.093; interaction F_(4,63)_=0.75, p=0.560) and pure intake of ethanol (**Supplemental Figure 2F**, 2way RM-ANOVA: sex F_(1,68)_=3.88, p=0.065; session F_(4,68)_=2.06, p=0.096; interaction F_(4,68)_=1.77, p=0.144) during the last 5 stable FR3 15min self-administration sessions.

## Sex differences in choice behavior after the BD procedure

We analyzed choice behavior for each option in the RGT, before and after ethanol, to include the sex factor (**Supplemental Figure 3**). The female rats were choosing the P1 option significantly more than the male rats before ethanol (Tukey p=0.008) (**Supplemental Figure 3A**, 2way RM-ANOVA: sex F_(1,8)_=4.919, p=0.057; treatment F_(1,8)_=0.00763, p=0.933; interaction (F_(1,8)_=7.019, p=0.029). The male rats chosed the P2 option significantly less after ethanol (Tukey p=0.01) (**Supplemental Figure 3B**, 2way RM-ANOVA: sex F_(1,8)_=0.0433, p=0.840; treatment F_(1,8)_=5.163, p=0.053; interaction F_(1,8)_=7.969, p=0.022). There were no effect of sex or the BD procedure on the P3 (**Supplemental Figure 3C**, 2way RM-ANOVA: sex F_(1,8)_=1.564, p=0.246; treatment F_(1,8)_=0.406, p=0.542; interaction F_(1,8)_=0.232, p=0.643) and P4 options (**Supplemental Figure 3D**, 2way RM-ANOVA: sex F_(1,8)_=1.586, p=0.243; treatment F_(1,8)_=0.053, p=0.824; interaction F_(1,8)_=0.2918, p=0.126) choice behavior.

# Supplementary Figures

**
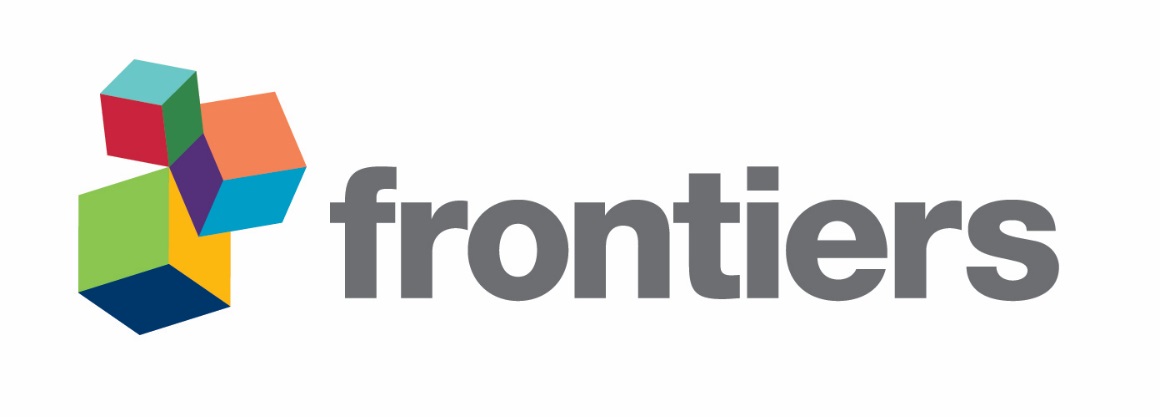
**

**SUPPLEMENTARY FIGURE 1 |** Other parameters during baseline sessions of the RGT. **(A)** No sex differences in the proportion of advantageous choices (P1 + P2) during the test sessions. Results are expressed as mean ± SEM of the proportion of advantageous choices over the total number of choices. **(B)** No sex differences in the number of trials made during the test sessions. Results are expressed as mean ± SEM of the total number of trials made. **(C)** Female rats made significantly more omissions than male rats. The dot line represents the threshold of allowed omitted responses for the animals during baseline test sessions. Results are expressed as mean ± SEM of the proportion of omitted trials over the total number of trials. *** p < 0.001. **(D)** No sex differences in the proportion of premature responses during the test sessions. Results are expressed as mean ± SEM of the proportion of premature responses over the total number of trials.

**SUPPLEMENTARY FIGURE 2** | Results from the Intermittent Access Two-Bottle Choice (IA2BC) and Operant Self-Administration (OSA) paradigm, in male and female rats. **(A)** Female rats had a significantly higher intake of ethanol by weight than male rats during the IA2BC sessions. Male rats, but not females, escalated their intake on the last session. Results are expressed as a mean ± SEM of the ethanol intake by weight during the 24 h sessions (g/kg/24h). *p<0.05, **p<0.01, *** p < 0.001 versus male rats, # p < 0.05 *versus* first session in male rats. $ p < 0.05 *versus* first session in female rats. **(B)** No sex differences in pure intake of ethanol during the IA2BC sessions. Male rats escalated their pure intake of ethanol while female did not. Results are expressed as a mean ± SEM of the total of pure intake of ethanol during the 24 h sessions (g/24 h). # p < 0.05, ### p < 0.001 *versus* first session in male rats. **(C)** Male rats had a significantly higher total fluid intake than female rats during the IA2BC sessions. Results are expressed as a mean ± SEM of the total fluid intake by weight during the 24h sessions (g/kg/24 h). *p<0.05, ** p < 0.01, versus female rats. **(D)** No sex differences in ethanol preference during the 24 h sessions. Male rats escalated their preference for ethanol while female did not. Results are expressed as a mean ± SEM of the proportion of ethanol consumed over total fluid consumed. ## p < 0.01, ### p < 0.001 *versus* first session in male rats. **(E)** No sex differences in ethanol intake by weight during the last 5 stable OSA FR3 15 min sessions. Results are expressed as a mean ± SEM of the ethanol intake by weight during the 15 minutes sessions (g/kg/15 min). **(F)** No sex differences in pure ethanol intake during the last 5 stable OSA FR3 15 min sessions. Results are expressed as a mean ± SEM of the total of pure ethanol intake during the 15 min sessions (g/15 min).

**SUPPLEMENTARY FIGURE 3** | Choice behavior for each option in the RGT, before and after ethanol. **(A)** Female rats chosed the P1 option significantly more in the RGT than male rats before ethanol. Results are expressed as mean ± SEM of the percent choice of the P1 option. **(B)** Male rats chosed the optimal P2 option significantly less in the RGT after ethanol. Results are expressed as mean ± SEM of the percent choice of the P2 option. **(C)** No effect of sex or treatment on the P3 option choice behavior. Results are expressed as mean ± SEM of the percent choice of the P3 option. **(D)** No effect of sex or treatment on the P4 option choice behavior. Results are expressed as mean ± SEM of the percent choice of the P4 option. * p <0.05, ** p < 0.01.

| Choices | P1 | P2 | P3 | P4 |
| --- | --- | --- | --- | --- |
| Reward trials  (# pellets and probability) | 1 (0.9) | 2 (0.8) | 3 (0.5) | 4 (0.4) |
| Punished trials (seconds and probability) | 5 (0.1) | 10 (0.2) | 30 (0.5) | 40 (0.6) |
| Hypothetical maximum pellets if option chosen throughout | 295 | 411 | 135 | 99 |

**Supplementary Table S1.** Modalities of reward and punishment in the RGT. Advantageous choices (P1 and P2) are associated with low immediate reward but also low probability of punishment, while disadvantageous choices (P3 and P4) are associated with high immediate reward but also high probability of punishment. If a rat were to choose only one option, then the greatest number of pellets possible would be with P2 (411, most optimal option), then P1 (295), P3 (135) and P4 (99, least optimal option).

| Figure number | Factor name | F,H or r values | *p*-value |
| --- | --- | --- | --- |
| Figure 1B. Sex differences in baseline RGT  (% correct trials)  2way RM-ANOVA | Sex  Session  Sex x Session | F_1,191_=2.899  F_24,191_=20.306  F_24,191_=1.180 | p=0.127  **p<0.001**  p=0.264 |
| Figure 1C. Sex differences in baseline RGT  (% choice)  2way RM-ANOVA | Sex  Option  Sex x Option | F_1,24_=0.346  F_3,24_=0.1.436  F_3,24_=5.740 | p=0.573  p=0.257  **p=0.004** |
| Figure 2A. RGT test after BD exposure  Males  2way RM-ANOVA | Treatment  Option  Treatment x Option | F_1,18_=26.761  F_3,18_=0.390  F_3,18_=2.694 | **p<0.001**  p=0.761  p=0.069 |
| Figure 2B. RGT test after BD exposure  Females  2way RM-ANOVA | Treatment  Option  Treatment x Option | F_1,24_=3.006  F_3,24_=2.363  F_3,24_=1.314 | p=0.117  p=0.093  p=0.290 |
| Figure 2C. RGT test after BD exposure  Advantageous choices P1+P2  2way RM-ANOVA | Sex  Treatment  Sex x Treatment | F_1,6_=1.902  F_1,6_=2.170  F_1,6_=0.380 | p=0.186  p=0.159  p=0.546 |
| Figure 2D. RGT test after BD exposure  Number of trials  2way RM-ANOVA | Sex  Treatment  Sex x Treatment | F_1,6_=0.725  F_1,6_=6.484  F_1,6_=0.643 | p=0.406  **p=0.021**  p=0.434 |
| Figure 2E. RGT test after BD exposure  Number of omissions  2way RM-ANOVA | Sex  Treatment  Sex x Treatment | F_1,6_=9.378  F_1,6_=3.722  F_1,6_=12.657 | **p=0.007**  p=0.071  **p=0.002** |
| Figure 2F. RGT test after BD exposure  Premature responses  2way RM-ANOVA | Sex  Treatment  Sex x Treatment | F_1,6_=0.824  F_1,6_=30.164  F_1,6_=5.697 | p=0.377  **p<0.001**  **p=0.029** |
| Figure 3A. Correlation between advantageous choices and ethanol intake in the IA2BC  Pearson correlation test | Males  Females | r=-0.3273  r=-0.1682 | p=0.3899  p=0.6422 |
| Figure 3B. Correlation between advantageous choices and ethanol preference in the IA2BC  Pearson correlation test | Males  Females | r=-0.3249  r=0.1291 | p=0.1056  p=0.7222 |
| Figure 3C. Correlation between advantageous choices and active lever presses in OSA  Pearson correlation test | Males  Females | r=-0.3288  r=-0.03515 | p=0.3876  p=0.9232 |
| Figure 3D. Correlation between advantageous choices and ethanol intake in OSA  Pearson correlation test | Males  Females | r=-0.3629  r=-0.09164 | p=0.3372  p=0.8012 |
| Figure 3E. Correlation between premature responses and active lever presses in OSA  Pearson correlation test | Males  Females | r=-0.02202  **r=0.7401** | p=0.9955  **p=0.0144** |
| Figure 3F. Correlation between premature responses and ethanol intake in the OSA  Pearson correlation test | Males  Females | r=-0.06348  **r=-0.6688** | p=0.8711  **p=0.0345** |
| Figure 4B. NOR test. Exploration time.  Males  2way RM-ANOVA | Object  Treatment  Group x Treatment | F_1,34_=211.055  F_1,34_=5.650E-030  F_1,34_=0.0203 | **p<0.001**  p=1.000  p=0.655 |
| Figure 4C. NOR test. Exploration time.  Females  2way RM-ANOVA | Object  Treatment  Group x Treatment | F_1,9_=38.124  F_1,9_=0.000  F_1,9_=0.349 | **p<0.001**  p=1.000  p=0.569 |
| Figure 4E. LDB test. Time in lit compartment.  2way RM-ANOVA | Sex  Treatment  Sex x Treatment | F_1,17_=8.383  F_1,17_=3.378  F_1,17_=0.0.000249 | **p=0.010**  p=0.084  p=0.988 |
| Figure 5B. Top panel. FSCV [DA]max  Males  2way RM-ANOVA | Group  Treatment  Group x Treatment | F_1,10_=0.00594  F_2,10_=24.715  F_2,10_=1.007 | p=0.442  **p<0.001**  p=0.399 |
| Figure 5B. Top panel. FSCV [DA]max  Females  2way RM-ANOVA | Group  Treatment  Group x Treatment | F_1,10_=0.605  F_2,10_=8.231  F_2,10_=1.780 | p=0.472  **p=0.008**  p=0.218 |
| Figure 5B. Middle panel panel. FSCV [DA]p  Males  2way RM-ANOVA | Group  Treatment  Group x Treatment | F_1,10_=0.116  F_2,10_=33.906  F_2,10_=1.328 | p=0.747  **p<0.001**  p=0.308 |
| Figure 5B. Middle panel. FSCV [DA]p  Females  2way RM-ANOVA | Group  Treatment  Group x Treatment | F_1,10_=1.828  F_2,10_=10.346  F_2,10_=1.387 | p=0.234  **p=0.004**  p=0.294 |
| Figure 5B. Bottom panel. FSCV Vmax  Males  2way RM-ANOVA | Group  Treatment  Group x Treatment | F_1,10_=3.787  F_2,10_=12.283  F_2,10_=1.156 | p=0.109  **p=0.002**  p=0.354 |
| Figure 5B. Bottom panel. FSCV Vmax  Females  2way RM-ANOVA | Group  Treatment  Group x Treatment | F_1,10_=0.949  F_2,10_=1.394  F_2,10_=0.0214 | p=0.375  p=0.292  p=0.979 |

**Supplementary Table S2.** Statistical analysis of the behavioral and FSCV datas.

|  |  | **Advantageous choices (%)** | **Number of trials** | **Omissions (%)** | **Premature responses (%)** | **[DA]max** | **[DA]p** | **Vmax** |
| --- | --- | --- | --- | --- | --- | --- | --- | --- |
| **EtOH intake IA2BC (g/kg/24h)** | Males | r=-0.3273 ; p=0.3899 | r=-0.4283 ; p=0.2501 | r=-0.7593 ; p=0.8461 | r=-0.2676 ; p=0.4863 | r=0.5313 ; p=0.1754 | r=0.5376 ; p=0.1694 | r=0.4764 ; p=0.2327 |
|  | Females | r=-0.1682 ; p=0.6422 | r=-0.2838 ; p=0.4268 | r=-0.1056 ; p=0.7716 | r=0.4046 ; p=0.2461 | r=0.286 ; p=0.5341 | r=0.2082 ; p=0.6542 | r=0.04378 ; p=0.9257 |
| **EtOH preference IA2BC (g/kg/24h))** | Males | r=-0.3249 ; p=0.1056 | r=-0.3315 ; p=0.3835 | r=0.1134 ; p=0.7714 | r=-0.5041; p=0.1665 | r=0.3771 ; p=0.3571 | r=0.4184 ; p=0.3023 | r=0.4841 ; p=0.2241 |
|  | Females | r=-0.1291 ; p=0.7222 | r=-0.4121 ; p=0.2367 | r=0.07182 ; p=0.8437 | r=0.4081 ; p=0.2417 | r=0.1571 ; p=0.7365 | r=0.1194 ; p=0.7987 | r=0.01595 ; p=0.9729 |
| **Active lever presses BD** | Males | r=-0.3288 ; p=0.3876 | r=-0.2749 ; p=0.4741 | r=0.5966 ; p=0.0899 | r=-0.002202 ; p=0.9955 | r=0.1307 ; p=0.7577 | r=0.1783 ; p=0.6727 | r=0.3009 ; p=0.469 |
|  | Females | r=-0.03515 ; p=0.0932 | r=-0.2596 ; p=0.4688 | r=-0.4275 ; p=0.2178 | **r=0.7401 ; p=0,0144** | r=-0.1709 ; p=0.7142 | r=0.2509 ; p=0.5874 | r=0.6576 ; p=0.1084 |
| **EtOH intake BD (g/kg/15min)** | Males | r=-0.3629 ; p=0.3372 | r=-0.3027 ; p=0.4285 | r=0,5613 ; p=0.1158 | r=0.06348 ; p=0.8711 | r=0.1433 ; p=0.735 | r=0.1839 ; p=0.6629 | r=0.2814 ; p=0.4996 |
|  | Females | r=-0.09164 ; p=0.8012 | r=-0.2689 ; p=0.4525 | r=-0.4526 ; p=0.1890 | **r=0.6688 ; p=0.0345** | r=-0.2797 ; p=0.5435 | r=0.08761 ; p=0.8518 | r=0.6295 ; p=0.1298 |
| **[DA]max** | Males | r=-0.1616 ; p=0.7023 | r=-0.4079 ; p=0.3158 | r=-0.2817 ; p=0.4992 | r=0.4489 ; p=0.2646 |  |  |  |
|  | Females | r=0.4678 ; p=0.2898 | r=0.6555 ; p=0.1099 | r=-0.5263 ; p=0.2249 | r=0.1670 ; p=0.7204 |  |  |  |
| **[DA]p** | Males | r=-0.103 ; p=0.8082 | r=-0.4847 ; p=0.2235 | r=-0.2598 ; p=0.5343 | r=0.3523 ; p=0.3921 |  |  |  |
|  | Females | r=0.6011 ; p=0.1534 | r=0.5334 ; p=0.2176 | r=-0.4828 ; p=0.2724 | r=0.4536 ; p=0.3067 |  |  |  |
| **Vmax** | Males | r=0.1356 ; p=0.7489 | r=-0.6072 ; p=0.1104 | r=-0.1429 ; p=0.7358 | r=0.0103 ; p=0.9807 |  |  |  |
|  | Females | r=0.5874 ; p=0.1655 | r=0.1333 ; p=0.7758 | r=-0.5778 ; p=0.1742 | r=0.7262 ; p=0.0646 |  |  |  |

**Supplementary Table S3.** Correlation analysis between all the parameters from the baseline RGT, the BD procedure and the FSCV data.
